# Substrate preference, RNA binding and active site versatility of *Stenotrophomonas maltophilia* nuclease SmNuc1, explained by a structural study

**DOI:** 10.1111/febs.17265

**Published:** 2024-10-03

**Authors:** Kristýna Adámková, Mária Trundová, Tomáš Kovaľ, Blanka Husťáková, Petr Kolenko, Jarmila Dušková, Tereza Skálová, Jan Dohnálek

**Affiliations:** ^1^ Institute of Biotechnology Czech Academy of Sciences Vestec Czech Republic; ^2^ Department of Biochemistry and Microbiology University of Chemistry and Technology Prague 6 Czech Republic; ^3^ Czech Technical University in Prague Czech Republic

**Keywords:** c‐di‐GMP cleavage, RNA, S1/P1 nuclease, *Stenotrophomonas maltophilia*, X‐ray crystallography

## Abstract

Nucleases of the S1/P1 family have important applications in biotechnology and molecular biology. We have performed structural analyses of SmNuc1 nuclease from *Stenotrophomonas maltophilia*, including RNA cleavage product binding and mutagenesis in a newly discovered flexible Arg74‐motif, involved in substrate binding and product release and likely contributing to the high catalytic rate. The Arg74Gln mutation shifts substrate preference towards RNA. Purine nucleotide binding differs compared to pyrimidines, confirming the plasticity of the active site. The enzyme–product interactions indicate a gradual, stepwise product release. The activity of SmNuc1 towards c‐di‐GMP in crystal resulted in a distinguished complex with the emerging product 5′‐GMP. This enzyme from an opportunistic pathogen relies on specific architecture enabling high performance under broad conditions, attractive for biotechnologies.

## Graphical abstract

Our structural analysis of the highly active SmNuc1 nuclease from opportunistic human pathogen *Stenotrophomonas maltophilia* brings new insights into the RNA binding and cleavage mechanism of the S1/P1 nuclease family. SmNuc1 has a distinctive highly mobile Arg74‐motif contributing to the active site. Mutagenesis and activity studies of the motif demonstrate its significant impact on substrate preference.

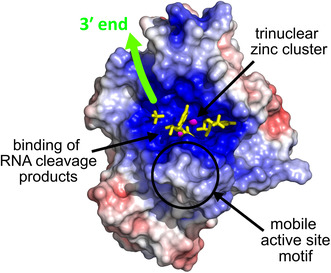

**Abbreviations**
1PE: pentaethylene glycol
5′‐AMP: 5′‐adenosine monophosphate
5′‐CMP: 5′‐cytidine monophosphate
5′‐dAMP(S): 2′‐deoxyadenosine 5′‐thiophosphate
5′‐GMP: 5′‐guanosine monophosphate
5′‐UMP: 5′‐uridine monophosphate
ADP: atomic displacement parameter
AU: asymmetric unit
c‐di‐GMP: cyclic diguanosine‐5′‐monophosphate
FOM: figure of merit
GOL: glycerol
LTD: lattice translocation defect
NBS1: nucleoside binding site 1
NFW: nuclease‐free water
PCR: polymerase chain reaction
PDB: protein data bank
PEG: polyethylene glycol
PGE: triethylene glycol
pGpG: 5′‐phosphoguanylyl‐(3′,5′)‐guanosine
Pi: phosphate ion
SmNuc1: S1/P1 nuclease from *Stenotrophomonas maltophilia*
SmNuc1R74K: mutant R74K of SmNuc1
SmNuc1R74Q: mutant R74Q of SmNuc1
WT: wild type

## Introduction

S1/P1 nucleases are a family of zinc‐dependent nucleases that were first isolated and characterised from *Lentinus edodes*, *Aspergillus oryzae* and *Penicillium citrinum* fungi [1, 2, 3]. The gene for this nuclease was found in several species from different kingdoms. The biological function has been determined for some nucleases belonging to plant and fungal species (e.g. TBN1 nuclease, P1 nuclease; [4]). There are also some studies on the biological role of nucleases from bacteria and protozoan parasites, but still without definite answers.

The S1/P1 nuclease SmNuc1 from the bacterium *Stenotrophomonas maltophilia* has recently been recombinantly expressed and characterised in our laboratory [5]. SmNuc1 is an RNA‐preferring nuclease with several interesting features, such as the ability to cleave c‐di‐GMP (a bacterial second messenger) at a significant catalytic rate, high activity within a wide range of temperature and pH, or one of the highest known catalytic rates of the whole family (*k*_cat_ of about 4100 s^−1^ for 3′‐nucleotidase activity).

Generally, the reaction rates of enzymes are typically between 1 and 10 000 molecules per second [6]. In the biological kingdom, catalase has the highest known turnover number of all enzymes cleaving up to 40 million molecules per second [7], followed by carbonic anhydrase with a catalytic rate of 10^4^ to 10^6^ s^−1^ [6]. The known turnover rates of nucleases are many times lower. Commonly used restriction endonucleases of type II (e.g. EcoRI, FokI) have a *k*_cat_ of only 0.002–0.02 s^−1^ [8, 9], and the commercially used Sma nuclease has a *k*_cat_ of 750 s^−1^ [10]. The high turnover number of SmNuc1, exceeding those of industrial nucleases, deserves an explanation.

Nucleases of the S1/P1 family are used in several applications exploiting the different cleavage abilities and substrate specificities. The S1 protection assay is based on the single‐strand preference of some S1/P1 nucleases and is widely used for nucleic acid analysis [4]. In contrast, dsDNase activity of CEL I and CEL II nucleases was used to detect single nucleotide mutations in heteroduplex DNA in mutation screening of the BRCA1 and BRCA2 genes [11, 12].

The cleavage mechanism of these nucleases has been proposed based on biochemical properties and structural analysis of the active site [13], which is conserved across the whole family. The mechanism of binding of longer chains of different substrates (dsDNA/ssDNA/RNA) is not yet understood in general, though in some cases, models were proposed [14]. This raises the question of what determines the substrate specificity and preferences of these nucleases and how this might be exploited in biotechnology [15].

In this work, we present structural analyses of the SmNuc1 nuclease and its complexes with RNA cleavage products, together with new insights into the cleavage mechanism and product release of S1/P1 nucleases.

## Results

This study brings experimental information on the structure of SmNuc1 nuclease from *S. maltophilia*, on RNA cleavage product interactions with this nuclease, including three structures with purine nucleotides, and three with pyrimidine nucleotides, and also a functional analysis of mutations in the newly discovered Arg74‐motif.

### Data quality

The diffraction limit of SmNuc1 structures ranges from 1.2 to 1.85 Å. The ligand‐free crystal structure of SmNuc1 (SmNuc1:free) contains one monomer of protein in asymmetric unit (AU), the rest of the solved structures contain two molecules in AU. The anisotropy correction applied to the SmNuc1:AMP structure successfully improved the behaviour of the structure during refinement. The presence of the lattice translocation defect (LTD) in SmNuc1:UMP led to a correction of intensities (Eqn 2, Data processing ) with translation vector **t**_d_ (0.4743, 0.0000, 0.5297) with fraction *k* = 0.05. After the correction the statistics *R*_work_, *R*_all_ and *R*_free_ decreased by 0.005, FOM and CC_all_ increased, geometric parameters improved, and no strong peaks were present in the difference electron density specific for LTD.

### Overall structure

**Fig. 1:**
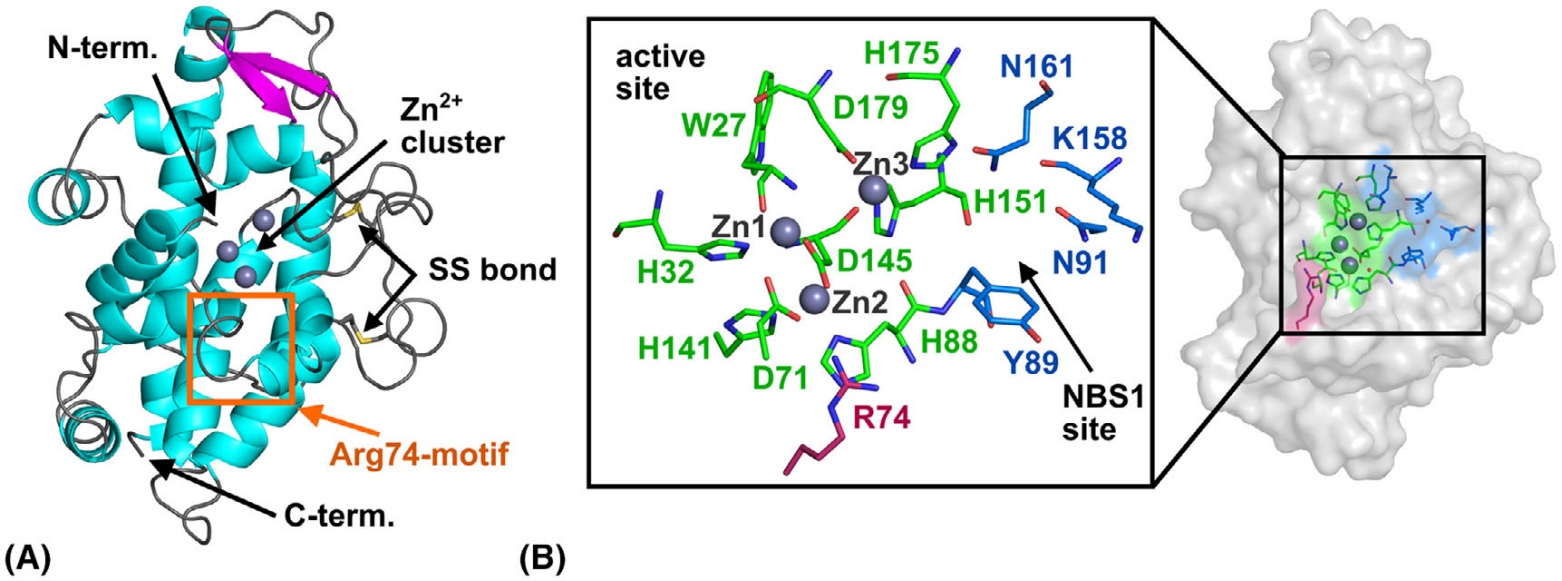
Overall structure of SmNuc1. (A) Cartoon representation of SmNuc1 coloured by secondary structure (α‐helices in cyan, β‐strands in magenta and loops in grey). The N‐terminal Trp27 is involved in the coordination of the zinc cluster. The Arg74‐motif is marked by the orange rectangle. (B) The active site of SmNuc1. The active site consists of three zinc ions (grey spheres) which are coordinated by nine residues (C atoms in green), residues of the NBS1 site (C in blue), and the stabilising complementary positive residue Arg74 (C in pink). Localization of the active site on the surface of SmNuc1 is highlighted. Graphics were created using pymol (Schrödinger, LLC, New York, NY, USA) and the SmNuc1:free structure (PDB: 8QJL).

**Fig. 2:**
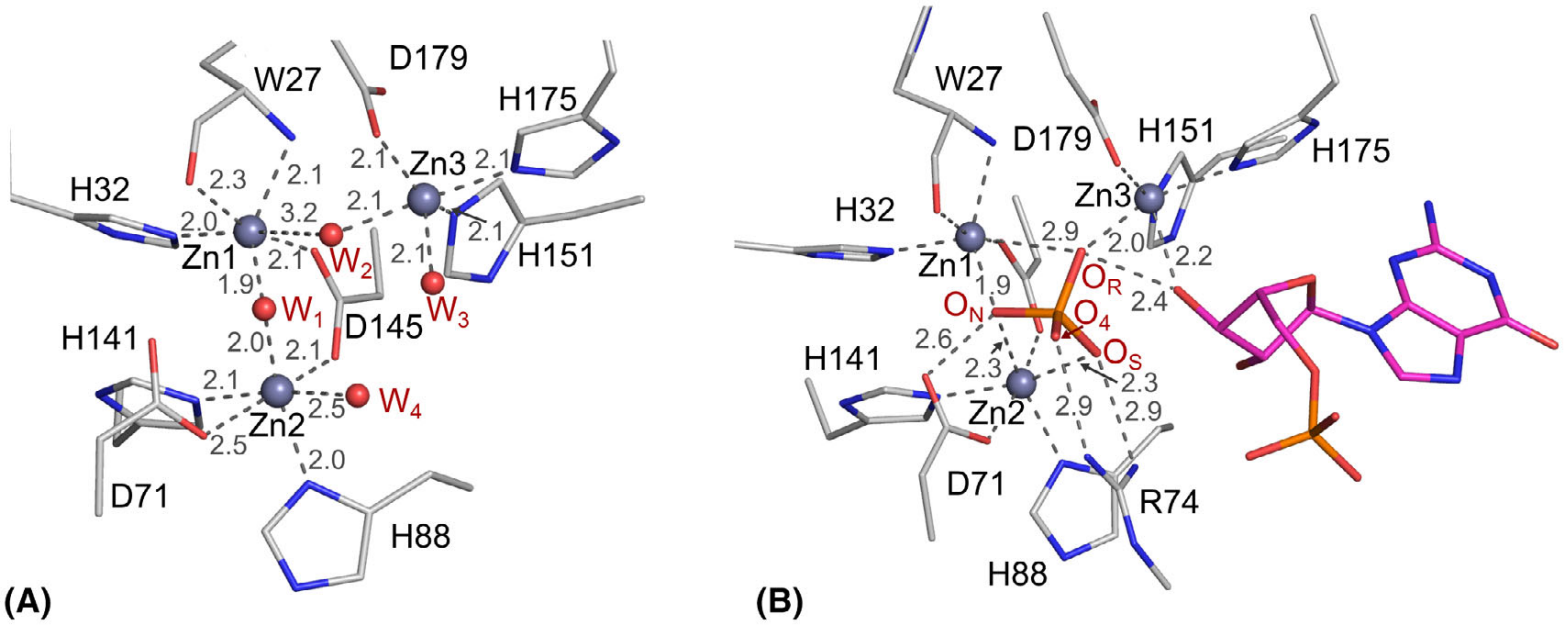
Zinc cluster of SmNuc1 and binding of phosphate ion. (A) Zinc cluster of the ligand‐free structure of SmNuc1 (PDB: 8QJL). Zinc ions Zn1, Zn2 and Zn3 (shown as grey spheres) are coordinated by the Trp27 main chain and side chains of eight residues (C atoms in grey). Water molecules W_1_, W_2_, W_3_ and W_4_ (red spheres) are coordinated by zinc ions. (B) Binding of the phosphate ion (Pi) in the structure of the SmNuc1:GMP complex (PDB: 8QJO). Oxygen O_N_ of Pi replaces the nucleophilic water W_1_, oxygen O_R_ replaces W_2_, and oxygen O_S_ replaces W_4_. W_3_ is replaced by the oxygen O3′ of the ribose moiety of the bound ligand 5′‐GMP (C in magenta). All contacts are shown as grey dashes and distances are in Å. Graphics were created using pymol (Schrödinger, LLC).

The overall structure of SmNuc1 resembles other S1/P1 nucleases with some distinct features. Mature SmNuc1 is an enzyme of 246 amino acids with a molecular weight of 28 kDa [5], composed mostly of α‐helices and stabilised by two disulfide bridges: Cys97‐Cys223 and Cys105‐Cys110 (Fig. 1A). The active site pocket consists of a trinuclear zinc cluster (Zn1, Zn2, Zn3) coordinated by nine residues: Trp27, His32, Asp71, His88, His141, Asp145, His151, His175 and Asp179 (Fig. 1B). Four water molecules (W_1_, W_2_, W_3_ and W_4_) are coordinated between zinc ions in the ligand‐free structure of SmNuc1:free (Fig. 2A). The nucleoside binding site 1 (NBS1), responsible for binding of the nucleobase and the ribose moiety of a substrate, is composed of Tyr89, Asn91, Lys158 and Asn161. The complementary positively charged residue, analogue to Lys68 of S1 nuclease [13] is Arg74. Positively charged residues, Arg and Lys, are mainly present on the protein surface in relatively high numbers (22 and 8, respectively, ~ 12% of amino acids) and contribute to the overall positive charge of SmNuc1 under neutral conditions (pI ~ 8.3; [5]).

### Binding of RNA cleavage products

#### Phosphate ion

**Fig. 3:**
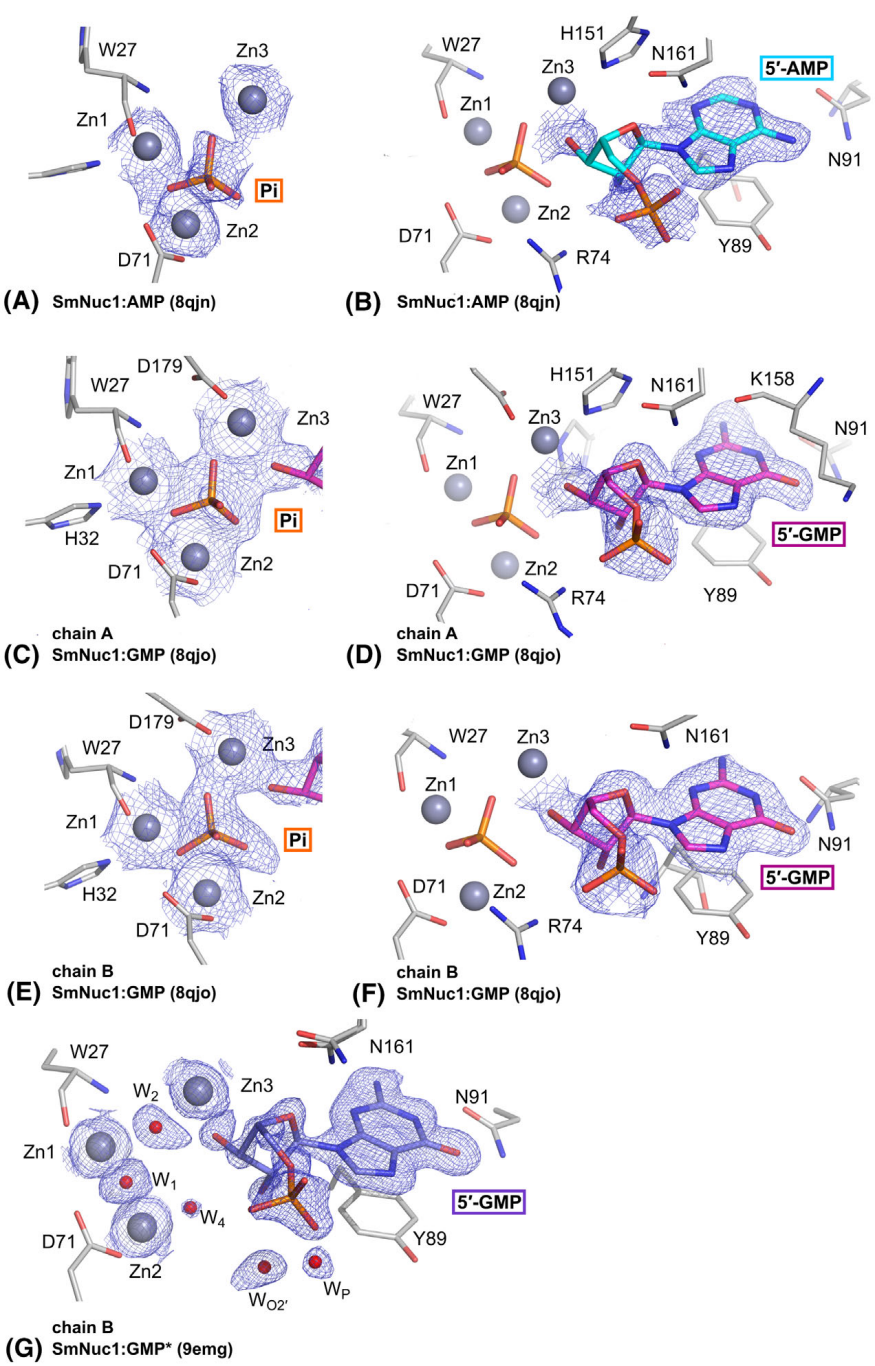
Ligand binding in the active site of SmNuc1:AMP (PDB: 8QJN), SmNuc1:GMP (PDB: 8QJO) and SmNuc1:GMP* (PDB: 9EMG) with 2m*F*_o_ − D*F*_c_ (blue mesh, [45]) composite omit map around ligands at 1σ level. (A) The active site of the SmNuc1:AMP complex with bound phosphate ion (P atom in orange) and (B) 5′‐AMP (C in cyan). (C) The active site of chain A of AU of the SmNuc1:GMP complex with bound phosphate ion (P in orange) and (D) 5′‐GMP (C in magenta). (E) The active site of chain B of AU of the SmNuc1:GMP complex with bound phosphate ion (P in orange) and (F) 5′‐GMP (C in magenta). (G) The active site of chain B of AU of the SmNuc1:GMP* complex (product of c‐di‐GMP cleavage) with bound waters and 5′‐GMP (C in violet). Graphics were created using pymol (Schrödinger, LLC).

A phosphate ion (Pi) is present in the active site of SmNuc1:AMP, SmNuc1:GMP, SmNuc1:UMP, and SmNuc1:CMPinh. In all these structures, it is in the position previously described as the inverted post‐cleavage state [13, 16]: O_N_ between Zn1 (1.9 Å) and Zn2 (2.3 Å), O_R_ in contact with Zn1 (2.9 Å) and Zn3 (2.0 Å), O_S_ interacting with Zn2 (2.3 Å) and the complementary residue Arg74 (only in the case of SmNuc1:GMP and SmNuc1:AMP). Oxygen O_4_ is stabilised by Arg74 (2.9 Å) and by Asp71 (3.2 Å to O^δ1^ and O^δ2^). O_N_, O_R_ and O_S_ replace the water molecules W_1_, W_2_ and W_4_, respectively, normally present in the unoccupied active site (Figs 2B and 3C).

#### Binding of purine nucleotides

**Fig. 4:**
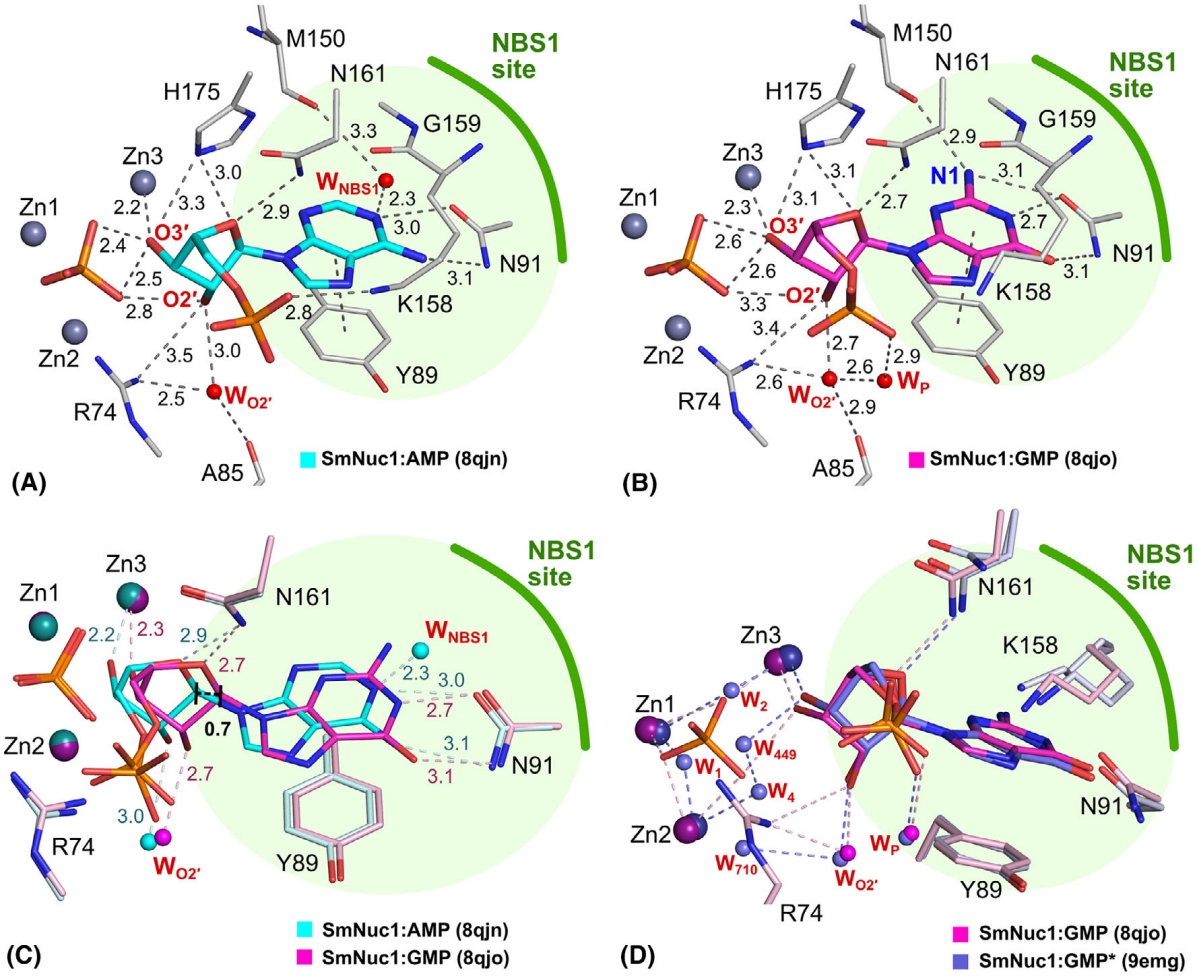
Binding modes of purine nucleotides in SmNuc1. (A) Binding of 5′‐AMP (C atoms in cyan) in chain B in the SmNuc1:AMP complex (PDB: 8QJN) and (B) binding of 5′‐GMP (C in magenta) in chain B in the SmNuc1:GMP complex (PDB: 8QJO). All ligand contacts are displayed as dashes and all distances are in Å. For simplicity, contacts of phosphate ions are not shown (these contacts are displayed in Fig. 2B). (C) Superposition of the active sites of the SmNuc1:AMP and SmNuc1:GMP complexes. SmNuc1:GMP is coloured in shades of magenta, and SmNuc1:AMP in shades of cyan. (D) Comparison of the active site of SmNuc1:GMP (C in magenta) and SmNuc1:GMP* (PDB: 9EMG, C in violet). SmNuc1:GMP is coloured in shades of magenta and SmNuc1:GMP* in shades of violet. Note the alternative conformations of Lys158 and Asn161 in SmNuc1:GMP* and the absence of Arg74 in the stabilisation of 5′‐GMP in the active site. Waters W_1_, W_2_, W_4_ and W_449_ replace oxygens of phosphate and W_710_ is in the previous position of Arg74. In both cases, oxygen O2′ of 5′‐GMP is stabilised by W_O2′_ and the phosphate moiety by interaction with W_P_. Graphics and the alignment, using the automated multi‐step superposition algorithm based on active site residues, were created using pymol (Schrödinger, LLC).

In the structures SmNuc1:AMP, SmNuc1:GMP and SmNuc1:GMP*, we observe the binding of purine nucleotides into the active site in the pose of expected products (Figs 3 and 4). The adenosine nucleobase is in π–π interaction with Tyr89 and the peptide bond Lys158‐Gly159, and it is in contact with Asn91 and with the water molecule W_NBS1_, which is a highly conserved water used for nucleobase coordination. The ribose moiety is in contact with Asn161, oxygen O3′ is in contact with Zn3 (2.2 Å) and the phosphate ion (Pi) present in the active site, and oxygen O2′ is stabilised by W_O2′_ and is also in contact with Pi. The phosphate moiety of the nucleotide is also in contact with Lys158.

The binding of 5′‐GMP in NBS1 of the SmNuc1:GMP structure is slightly different from that of 5′‐AMP. The nucleobase guanosine is in this comparison shifted out of the zinc cluster (0.7 Å) and stabilised by Asn91 through the hydroxy group (O6) on atom C6 of the nucleobase (3.1 Å to Asn91N^δ2^), amino group (N1 atom, 3.1 Å to Asn91O^δ1^) and atom N2 (2.7 Å to Asn91O^δ1^). The amino group (N1) of the adenosine nucleobase replaces the water molecule W_NBS1_ in the NBS1 site. The ribose and phosphate moieties of the nucleotide bind similarly to 5′‐AMP described above but are shifted out of the zinc cluster.

In the SmNuc1:GMP* structure, the active site has some differences compared to the SmNuc1:GMP complex structure. Residues Lys158 and Asn161 have alternative conformations and Arg74 is not in contact with the active site and is replaced by W_710_. The zinc cluster is occupied by waters W_1_, W_2_ and W_4_, and also by W_449_, which is in contact with O3′ of the ribose moiety. However, the position of 5′‐GMP in the NBS1 site is the same as in the SmNuc1:GMP structure.

#### Binding of pyrimidine nucleotides

**Fig. 5:**
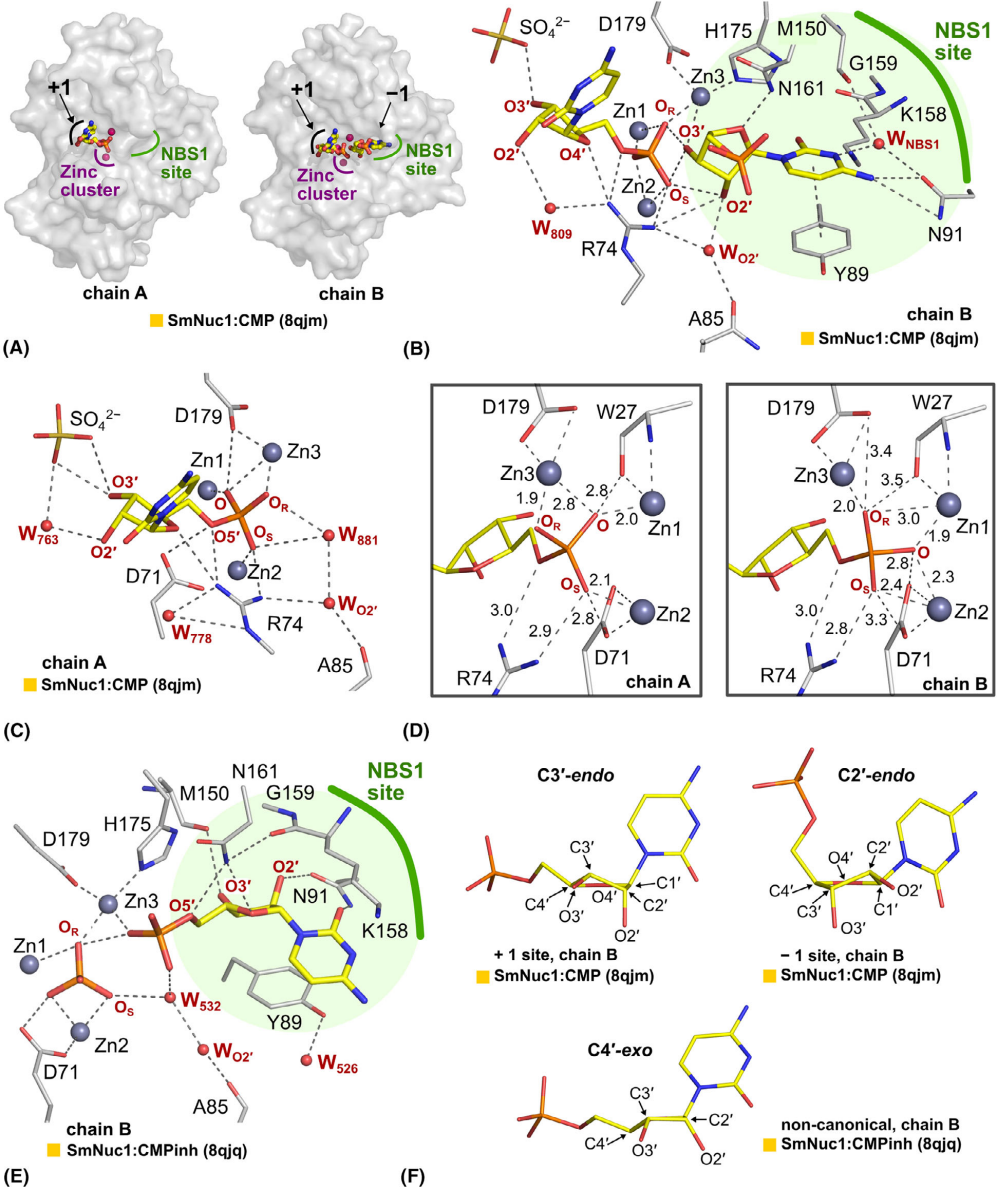
Binding modes of 5′‐CMP nucleotides in SmNuc1 structures. (A) Schematic representation of positions occupied by 5′‐CMP (+1 site and − 1 site) in the complex SmNuc1:CMP (PDB: 8QJM). (B) Binding of 5′‐CMP in chain B of AU of the SmNuc1:CMP complex. 5′‐CMP (C atoms in yellow) binds in the −1 site and +1 site mimicking the post‐cleavage state of a dinucleotide. H‐bonds and the interactions are marked by grey dashes. (C) Binding of 5′‐CMP in the +1 site of chain A of the SmNuc1:CMP complex. (D) The comparison of binding of phosphate moiety in the +1 site of chain A and B of the SmNuc1:CMP complex. Phosphate moiety is rotated in the zinc cluster and binds in two binding modes – inverted post‐cleavage state (chain B) and state of the leaving product (chain A). (E) Non‐canonical binding mode of 5′‐CMP in the NBS1 site of chain B of SmNuc1:CMPinh complex (PDB: 8QJQ). Note the position of the ribose moiety in the site normally occupied by the nucleobase. (F) Conformation of the ribose moiety of 5′‐CMP in the structures of the SmNuc1 complexes. In the +1 site the C3′‐*endo* conformation of the ribose moiety is preferred (chain B, SmNuc1:CMP). In the −1 site the ribose moiety has a C2′‐*endo* conformation (chain B, SmNuc1:CMP), whereas the C3′‐*endo* conformation is not possible in this site due to steric constraints. In the non‐canonical binding position of 5′‐CMP (SmNuc1:CMPinh complex), the ribose moiety is in C4′‐*exo* conformation. Graphics were created using pymol (Schrödinger, LLC). All ligand contacts are displayed as dashes. Distances in (D) are in Å.

5′‐CMP was observed in two structures in three positions with respect to the SmNuc1 active site and in total in four different conformations realised (Fig. 5A–E). The SmNuc1:CMP complex has two molecules in AU. In the active site of the first molecule, one 5′‐CMP binds to NBS1 (−1 position) and another 5′‐CMP in the +1 position of the nucleotide with respect to the cleaved phosphodiester bond (Fig. 5A). This is similar to 5′‐dCMP in the structure of S1 nuclease (PDB: 5FBF, [13]). In the NBS1 site (−1 site) of SmNuc1:CMP, the nucleobase is in π–π interaction with the aromatic side chain of Tyr89 and the peptide bond of Lys158‐Gly159, stabilised by Asn91 and the water molecule W_NBS1_. According to *PROSIT* (https://cactus.nci.nih.gov/prosit/; [17]) the ribose moiety is in the C2′‐*endo* conformation (Fig. 5F) as in the S1:CMP complex of S1 nuclease [16]. Oxygen O2′ of the ribose moiety is stabilised by the highly conserved water W_O2′_ (2.7 Å), oxygen O3′ is in contact with Zn3 (3.1 Å) and the phosphate moiety is positioned out of the active site pocket and oxygen O4′ is in contact with Asn161N^δ2^ (2.6 Å). Oxygen O3′ of the ribose moiety is also in contact with oxygen O_S_ (2.8 Å) and O_R_ (2.9 Å) of the 5′‐CMP nucleotide bound in +1 position and similarly oxygen O2′ with O_S_ (3.1 Å). The nucleobase of the mononucleotide in the +1 position is located outside of the active site pocket with no close contact with any protein residue. The entire nucleotide binds to the active site through the binding of its phosphate moiety to the zinc cluster, through the interaction of the ribose moiety's O4′ atom with Arg74N^η1^ and water molecules. The ribose moiety is in the C3′‐*endo* conformation as calculated in *PROSIT* (Fig. 5F). The phosphate moiety binds in the inverted post‐cleavage binding mode, where the oxygen O_S_ is in contact with Zn2 (2.4 Å), the oxygen O_4_ is located between Zn1 (1.9 Å) and Zn2 (2.3 Å) and O_R_ is in contact with Zn3 (2.0 Å) (Fig. 5B).

**Fig. 6:**
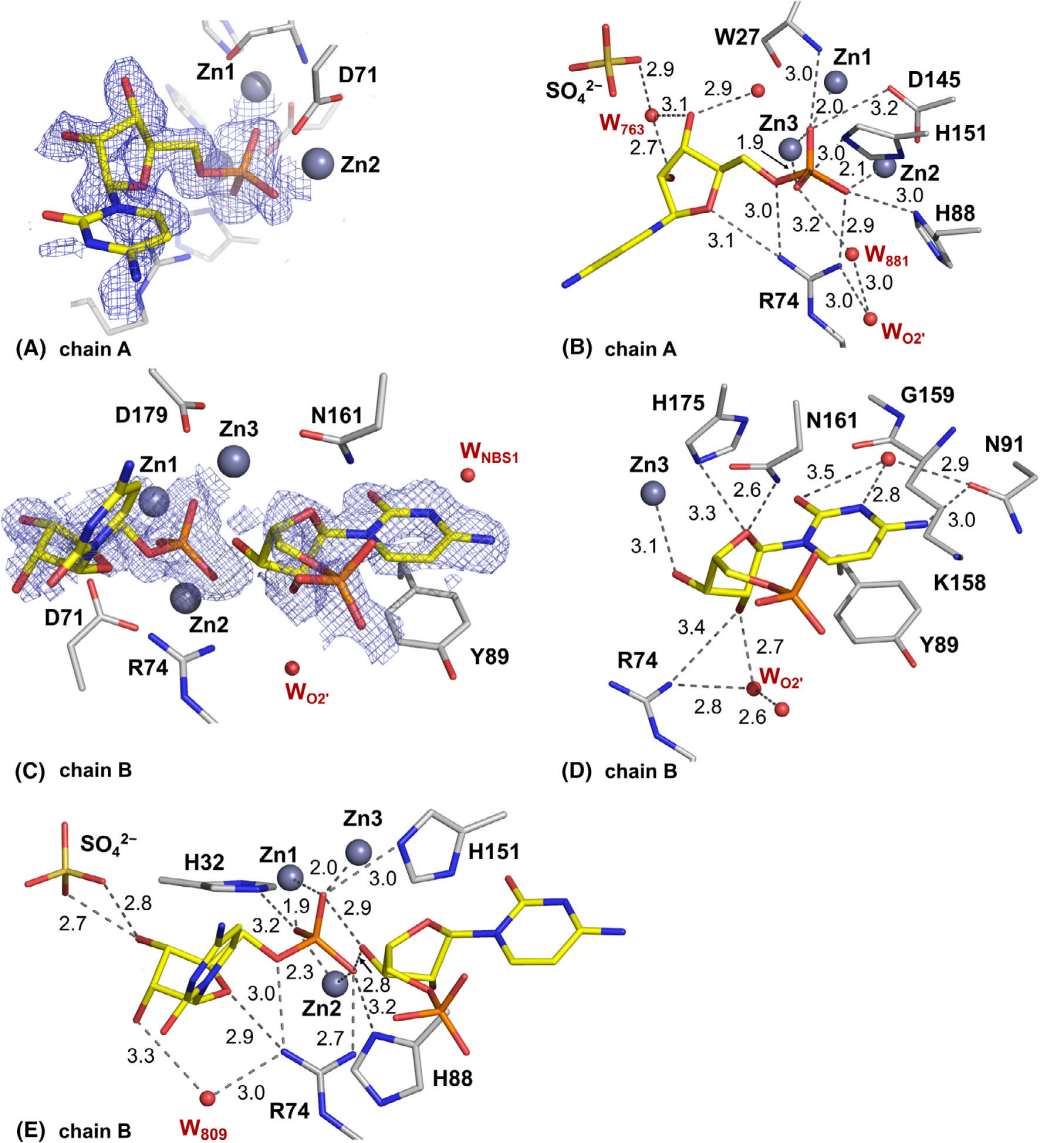
Binding of 5′‐CMP in the active site of the SmNuc1:CMP complex (PDB: 8QJM). (A) The active site of chain A of AU with 2m*F*_o_ − D*F*_c_ composite omit map at 1σ level (blue mesh; [45]) around 5′‐CMP (C atoms in yellow) bound in +1 position with respect to the cleaved phosphodiester bond, and (B) with displayed H‐bonds and O–Zn distances between 5′‐CMP, zinc ion Zn3, waters, and residues of the active site (grey dashes). (C) Active site of chain B with composite omit map 2m*F*_o_ − D*F*_c_ at 1σ level (blue mesh) around two molecules of 5′‐CMP (C in yellow) with (D) displayed H‐bonds and O–Zn distances of 5′‐CMP bound to NBS1, and (E) all contact distances of 5′‐CMP bound to +1 site. All distances are in Å. Graphics were created using pymol (Schrödinger, LLC).

Interestingly, in the second molecule in AU of SmNuc1:CMP, 5′‐CMP is found only in the +1 position and binds differently than previously described. The phosphate moiety is rotated, and O_S_ is in contact with Zn2 (2.0 Å), O with Zn1 (2.0 Å) and O_R_ with Zn3 (1.9 Å) (Figs 5C,D and 6).

**Fig. 7:**
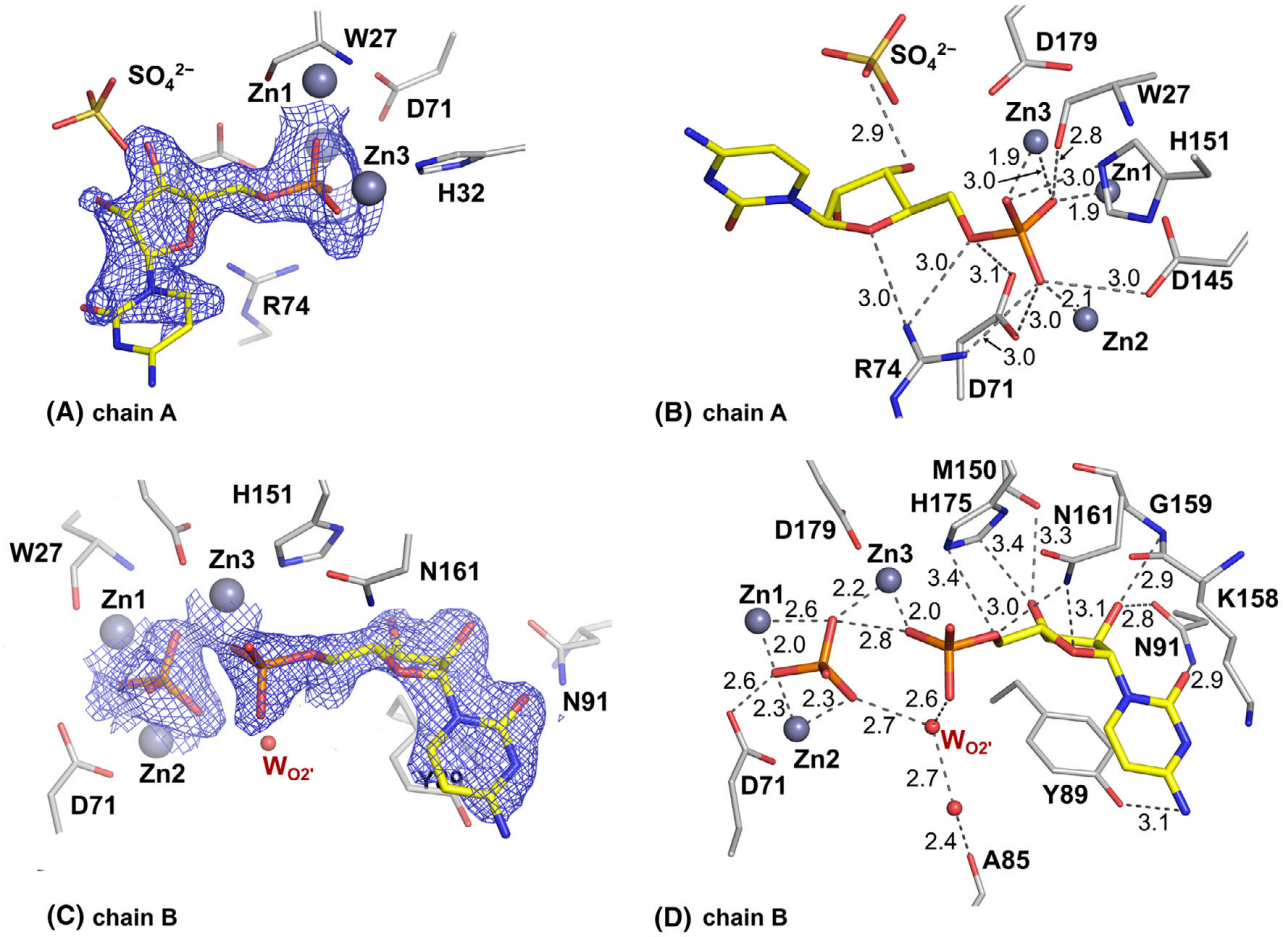
Binding of 5′‐CMP in the active site of the SmNuc1:CMPinh complex (PDB: 8QJQ). (A) Active site of chain A of AU with 2m*F*_o_ − D*F*_c_ composite omit map at 1σ level (blue mesh; [45]) around 5′‐CMP (C atoms in yellow) bound to +1 site with (B) displayed H‐bonds and O–Zn distances between ligand and residues of the active site (grey dashes). (C) Active site of chain B of AU with 2m*F*_o_ − D*F*_c_ composite omit map at 1σ level (blue mesh) around Pi (P in orange) and 5′‐CMP (C in yellow) bound in an inhibitory binding mode with (D) displayed H‐bonds and O–Zn distances of Pi and 5′‐CMP bound to the NBS1 site. All distances are in Å. Graphics were created using pymol (Schrödinger, LLC).

The structure of SmNuc1:CMPinh reveals a new possible binding mode of 5′‐CMP to the active site of nucleases from the S1/P1 family (Fig. 5C). The phosphate ion is located in the zinc cluster in the previously described inverted binding mode. The phosphate moiety of the nucleotide 5′‐CMP is in contact with Zn3 (2.0 Å) and this phosphate ion (2.8 Å to O_R_) and in contact with a stabilising water molecule W_532_. The ribose part of the nucleotide is in the C4′‐*exo* conformation (*PROSIT*; https://cactus.nci.nih.gov/prosit/; [17]), is flipped by ~ 180° compared to the canonical binding and binds to the area normally reserved for nucleobase. Oxygens O4′ and O5′ are stabilised by Asn161 (3.1 and 3.0 Å to atom N^δ2^) and O2′ is in contact with Asn91 (2.9 Å to atom O^δ1^). The nucleobase is bent towards the Tyr89 side of the NBS1 site and is stabilised through an OH–π interaction with this residue [18] (Figs 5E,F and 7).

**Fig. 8:**
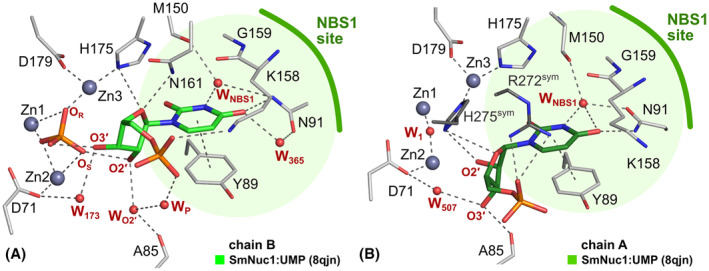
Binding of 5′‐UMP in the SmNuc1:UMP complex (PDB: 8QJN). (A) Binding of 5′‐UMP in chain B of AU of SmNuc1:UMP (C atoms of 5′‐UMP in green). (B) Binding of 5′‐UMP in chain A blocked by contact with the C terminus of chain A of the symmetry‐related molecule. Residue Arg272^sym^ and the His‐tag residue His275^sym^ (C in dark grey) are from the symmetry‐related molecule with translation vector in fractional coordinates **t** (*x* + 1, *y*, *z*). Selected interaction contacts are displayed as dashes. Graphics were created using pymol (Schrödinger, LLC).

**Fig. 9:**
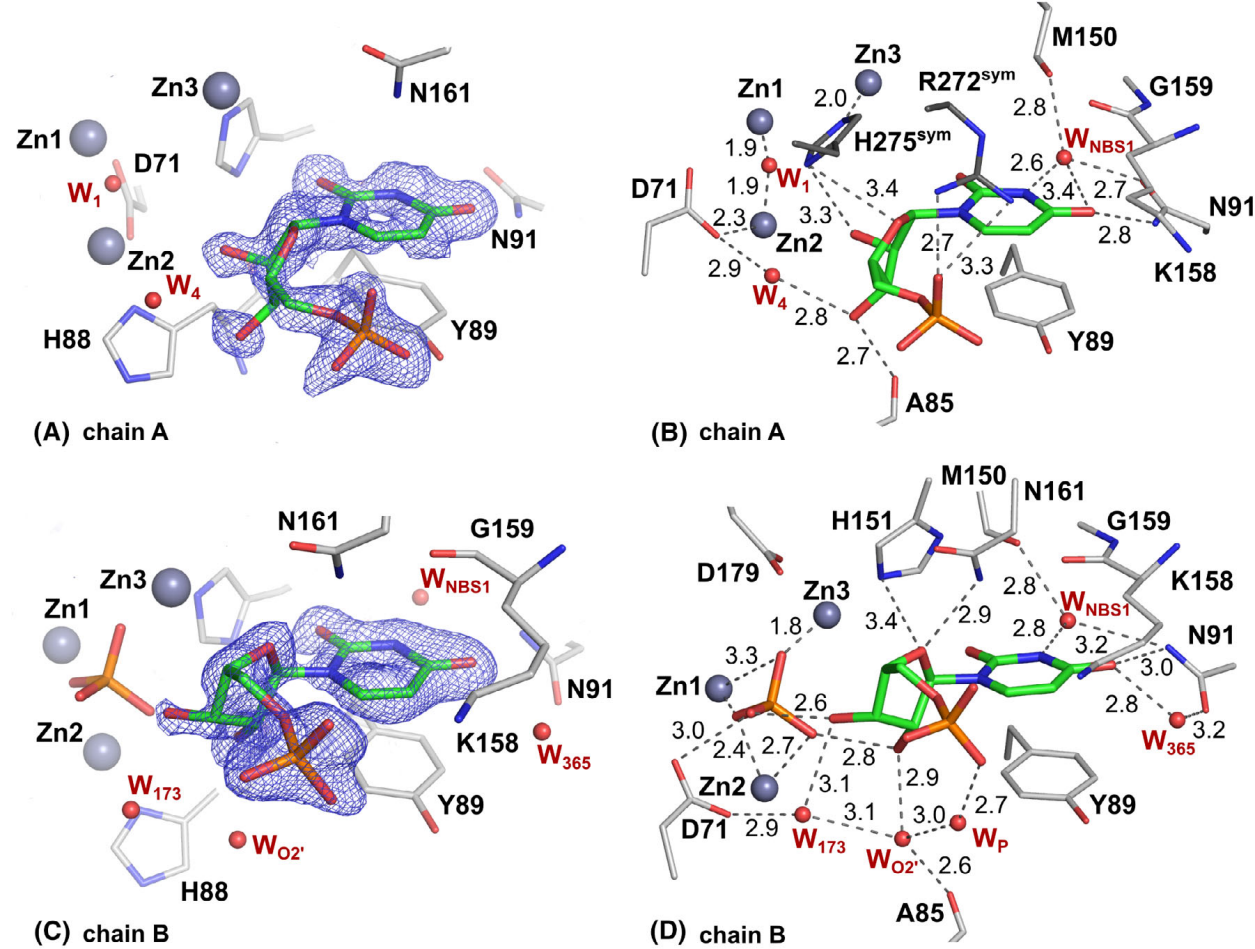
Binding of 5′‐UMP in the active site of the SmNuc1:UMP complex (PDB: 8QJP). (A) Active site of chain A of AU with 2m*F*_o_ − D*F*_c_ composite omit map at 1σ level (blue mesh; [45]) around 5′‐UMP (C atoms in green) with (B) displayed H‐bonds and O–Zn distances between 5′‐UMP, zinc ion Zn3, waters, and residues of the active site (grey dashes). (C) Active site of chain B of AU with 2m*F*_o_ − D*F*_c_ composite omit map at 1σ level (blue mesh) around 5′‐UMP (C in green) with (D) displayed H‐bonds and O–Zn distances of 5′‐UMP bound to the NBS1 site. All distances are in Å. Graphics were created using pymol (Schrödinger, LLC).

**Fig. 10:**
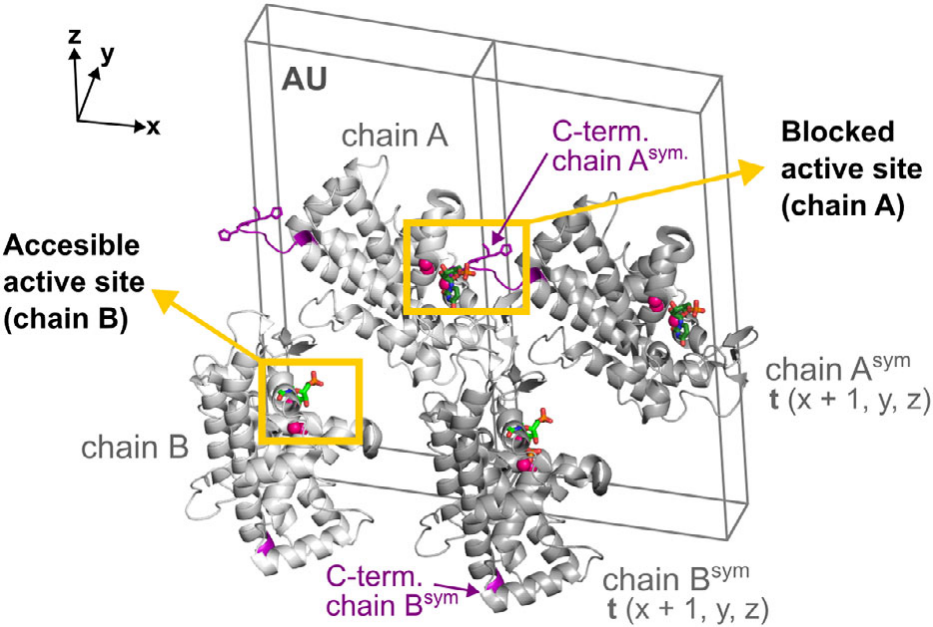
Crystal packing of the SmNuc1:UMP complex with blocked active site of chain A and accessible active site of chain B. There are two molecules of SmNuc1 in AU – (chain A and chain B) shown as light grey cartoon. Symmetry‐related molecules (chain A^sym^ and chain B^sym^) with translation vector **t** (*x* + 1, *y*, *z*) (fractional coordinates) are shown as dark grey cartoon. C‐termini of all displayed chains are highlighted in purple. Zinc ions are displayed as pink spheres, 5′‐UMP molecules are shown as sticks (C atoms in green). Graphics were created using pymol (Schrödinger, LLC).

**Fig. 11:**
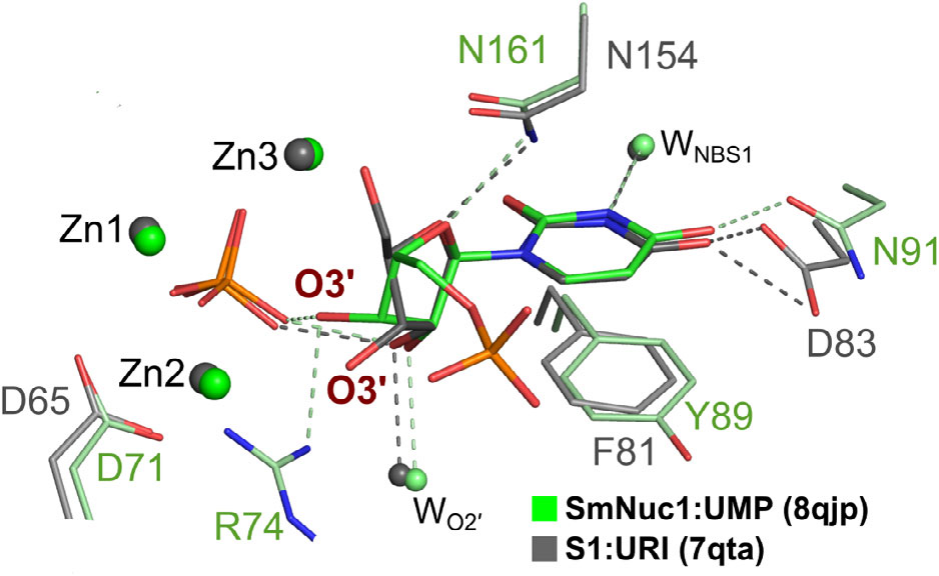
The comparison of binding of 5′‐UMP (C atoms in green) in the active site of the SmNuc1:UMP complex (C in light green; PDB: 8QJP) and uridine (URI, C in dark grey) in the active site of the S1:URI complex (C in grey; PDB: 7QTA, [13]). Important contacts are shown as dashes. Graphics were created using pymol (Schrödinger).

The ribose moiety of 5′‐UMP in the SmNuc1:UMP complex is slightly shifted out, away from the zinc cluster; atom O4′ at 2.9 Å from Asn161N^δ2^ and O3′ is not in contact with Zn3 but flipped out and stabilised by water W_173_ and in contact with one oxygen of the phosphate ion (2.6 Å) present in the active site zinc cluster (Fig. 8A). Interestingly, in the second chain in AU (designated chain A for technical reasons), the active site of the protein is complemented by residues from the C‐terminal part of the symmetry‐related molecule. His275^sym^ binds to Zn3 (2.0 Å to atom N^ε2^), causing a novel inhibitory binding mode of 5′‐UMP in the NBS1 site, where the whole nucleotide is rotated out of the zinc cluster. The ribose moiety and phosphate are stabilised by the side chains of residues Arg272^sym^ and His275^sym^. Oxygen O3′ replaces W_O2′_ (2.7 Å to Ala85O) and the nucleophile W_1_ is present between Zn1 and Zn2 (Figs 8 and 9). As the residues involved in ligand coordination belong to the His‐tag, we suggest that this binding mode is a relic of crystallisation (Fig. 10). Arg74 is not involved in coordination of the ligands in either case. Otherwise, the nucleobase of 5′‐UMP occupies the NBS1 site similarly to 5′‐CMP in the SmNuc1:CMP complex and to uridine in the S1 nuclease complex structure (PDB: 7QTA, [16]). The position of the ribose moiety, however, differs, specifically in the case of oxygen O3′ (Fig. 11).

### Arg74‐motif

**Fig. 12:**
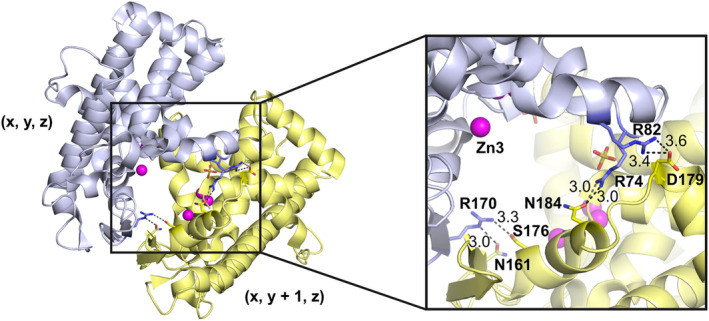
Crystal contacts in the SmNuc1:free structure involving the Arg74‐motif. The original molecule (*x*, *y*, *z*) is shown as grey cartoon with contact residues as sticks and the (*x*, *y* + 1, *z*) symmetry‐related molecule is shown as yellow cartoon with contact residues as sticks. Zinc ions are shown as spheres and coloured in pink. Contacts are shown as dashes and distances are in Å. Graphics were created using pymol (Schrödinger, LLC).

In the structures SmNuc1:free, SmNuc1:CMP and SmNuc1:UMP, we can see different states of the region between Asp71 and Tyr89, further called an Arg74‐motif. In the SmNuc1:free structure, Arg74 is flipped out of the active site and in contact with a symmetry‐related molecule (Fig. 12). After binding of 5′‐CMP in the active site of SmNuc1 (SmNuc1:CMP, PDB: 8QJM) Arg74 is in contact with ligands bound to the active site and fulfils its function as a complementary positively charged residue, coordinating nucleotides in position −1 in the NBS1 site directly by contact of atom Arg74‐N^η2^ with O2′ of the ribose moiety (3.4 Å), through the water molecule W_O2′_ (2.8 Å), and also coordinating nucleotide in the +1 position (Arg74N^η2^‐O_S_, 2.7 Å, Arg74N^η1^‐O4′, 2.9 Å, and Arg74N^η1^‐O5′, 3.0 Å) (Fig. 5B). In the SmNuc1:UMP structure, Arg74 is in an intermediate position between the zinc cluster (Arg74‐N^η1^ 9.9 Å away from Zn2). There is also an open conformation as in SmNuc1:free (Arg74‐N^η1^ 14.1 Å away from Zn2), without any contact with any other amino acid residue or ligand.

**Fig. 13:**
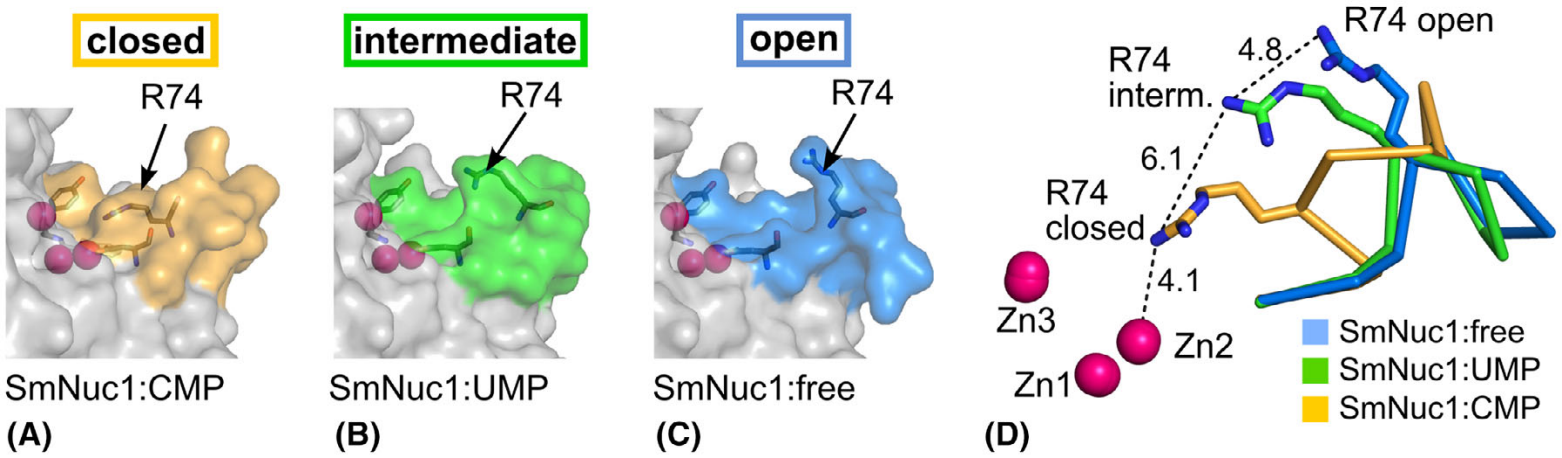
Different states of the Arg74‐motif (Asp71‐Tyr89) in the SmNuc1 structures. (A) Detail of SmNuc1:CMP (PDB: 8QJM) with the closed Arg74‐motif (surface coloured in orange, Asp71, Arg74 and Tyr89 in sticks). (B) Detail of SmNuc1:UMP (PDB: 8QJP) with the Arg74‐motif in the intermediate position (surface coloured in green, Asp71, Arg74 and Tyr89 in sticks). (C) Detail of SmNuc1:free (PDB: 8QJL) with the open Arg74‐motif (blue surface). Arg74 (shown as sticks) is flipped out. (D) Superposition of the SmNuc1:free, SmNuc1:UMP and SmNuc1:CMP structures with different positions of Arg74. Zinc ions are shown as spheres and coloured in pink. Distances in Å show the shift of the guanidium group of Arg74. The alignment was calculated based on C^α^ atoms, and all graphics were generated using pymol (Schrödinger, LLC).

The Arg74‐motif border residues Asp71 and Tyr89 are essential for the nucleolytic activity [4], and their position is very rigid. The most significant positional differences of the Arg74‐motifs in our structures are seen for the region Leu73‐Gly79. For Leu73C^α^ and Gly79C^α^ the difference in position between the open motif (SmNuc1:free) and the closed motif (SmNuc1:CMP) is ~ 1 Å. The most extreme difference in position is seen for Lys76C^α^ (5.8 Å). The closed (ligand bound), intermediate and open positions of the Arg74‐motif are shown in Fig. 13.

### Impact of Arg74 mutation on SmNuc1 activity

**Fig. 14:**
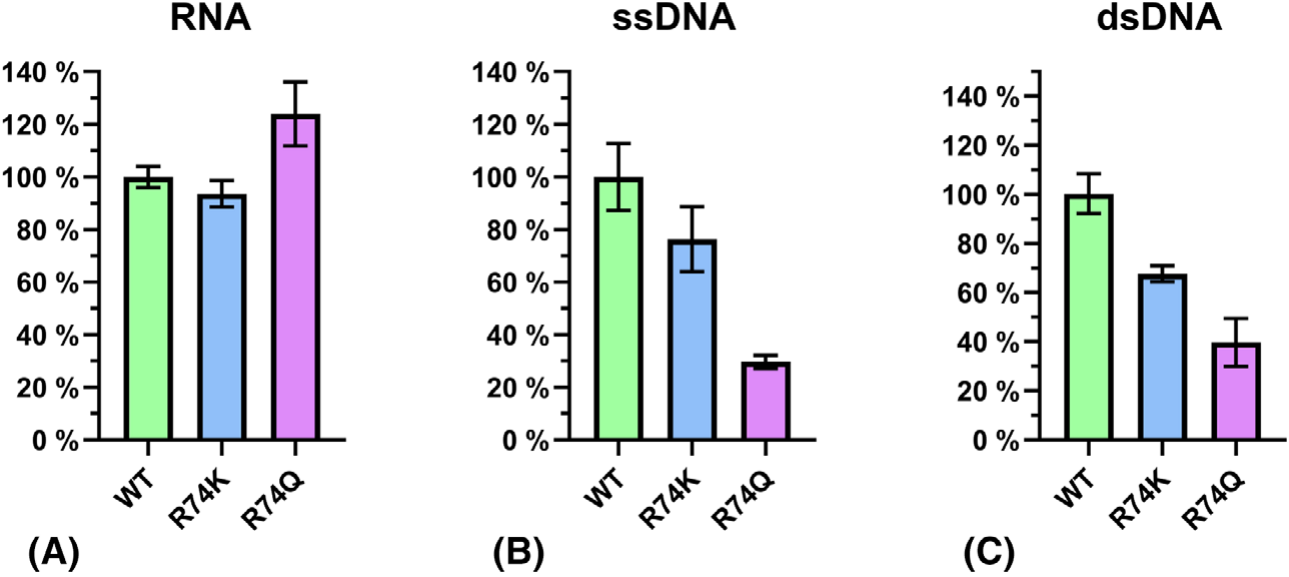
Comparison of the nucleolytic activity of SmNuc1WT (green) with SmNuc1R74K (blue) and SmNuc1R74Q (purple) mutants towards (A) RNA, (B) ssDNA, and (C) dsDNA substrates. All reactions were performed in triplicates at pH 7. The measured activities (including standard deviations) were converted into percentages of SmNuc1WT specific activity towards the indicated substrate type (58 550 ± 2330 U·μg^−1^ for RNA, 71 000 ± 8910 U·μg^−1^ for ssDNA, and 2150 ± 170 U·μg^−1^ for dsDNA). One unit (U) of nuclease activity was defined as change of absorbance of 0.001 at 260 nm in 1 cm path per 1 min [23]. Results were processed and graphs were produced using the graphpad prism8 software version 8.2 (GraphPad Software, Boston, MA, USA).

To further analyse the effect of Arg74 on the activity and the substrate preference of SmNuc1, and to explain the purpose of the Arg74‐motif structural changes, Arg74 was mutated to Lys and Gln. The sequences of the SmNuc1R74K and SmNuc1R74Q mutants were confirmed by mass spectrometry. A comparison of the nucleolytic activity of SmNuc1WT and SmNuc1R74K towards RNA substrate revealed no significant difference, and for SmNuc1R74Q there is even a slight increase in activity. However, when ssDNA and dsDNA were used as substrates, there was a significant decrease in the activity of both the SmNuc1R74K mutant (to 80% for ssDNA and to 70% for dsDNA) and the SmNuc1R74Q mutant (to 25% for ssDNA and to 40% for dsDNA) when compared to SmNuc1WT (Fig. 14).

## Discussion

### SmNuc1 nuclease

The structure of the SmNuc1 nuclease is the first 3D structure of a bacterial member of the S1/P1 family. The overall fold is similar to other known nucleases in this family with r.m.s.d. values calculated between C^α^ atoms of SmNuc1:free and the structurally closest representatives (using *PDBeFold* http://www.ebi.ac.uk/msd‐srv/ssm; [19]) of 1.49 Å for AtBFN2 (PDB: 4CXP, [14]), 1.56 Å for TBN1 nuclease (PDB: 4JDG, [20]), 1.64 Å for P1 nuclease (PDB: 1AK0, [21]) and 1.73 Å for S1 nuclease (PDB: 5FB9, [13]). However, there are some distinctive features of this bacterial enzyme, including the active site and its nearest surroundings. For the first time, we observe stabilisation of the phosphate moiety of the ligand in the −1 position not only by structural waters, but also by Lys158 (in SmNuc1:UMP and SmNuc1:AMP). SmNuc1 is the first representative of this family with a mobile motif containing the conserved basic residue involved in interactions with substrates and products.

### Binding of purine nucleotides

**Fig. 15:**
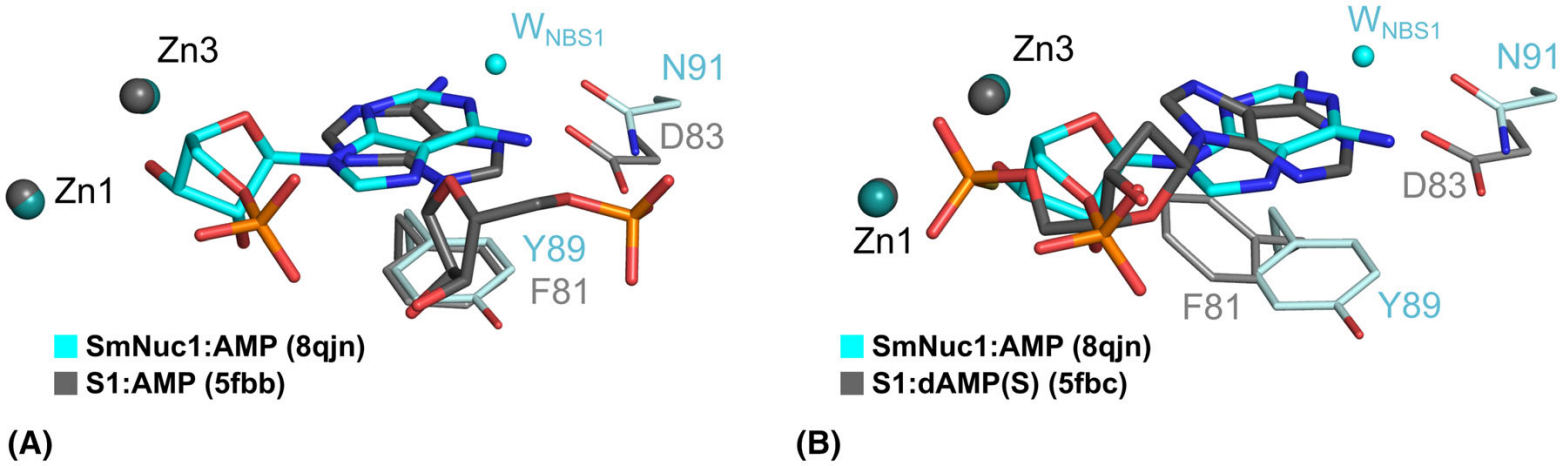
The comparison of binding of the purine nucleotide 5′‐AMP in SmNuc1 (C atoms in cyan) and adenosine nucleotides in structures of S1 nuclease complexes. (A) Alignment of 5′‐AMP from the SmNuc1:AMP structure (C in cyan) with 5′‐AMP (C in grey; PDB: 5FBB, [13]) bound to S1 nuclease in the inhibitory/inverted binding mode and (B) with 5′‐dAMP(S) (C in grey, PDB: 5FBC, [13]) bound in the remodelled NBS1 in an inhibitory position. Zinc cluster (spheres) and the displayed part of the NBS1 site are coloured light blue for SmNuc1:AMP and grey for the S1 nuclease structures. Alignments of the active site residues were calculated using an algorithm implemented in pymol (Schrödinger, LLC), used for generation of graphics.

The binding of purine nucleotides in the canonical positions (consistent with nucleic acid cleavage) confirmed the ability of these nucleases to bind all five standard nucleobases and cleave both DNA and RNA without significant preference for base. Before our study, only two positions of purine nucleotides in the active site had been observed in the whole family of S1/P1 nucleases, but both represented a non‐functional/inhibitory binding mode: the inverted deep binding mode of 5′‐AMP (PDB: 5FBB; [13]; Fig. 15A) and the site‐remodelling binding mode of 2′‐deoxyadenosine 5′‐thiophosphate (5′‐dAMP(S); PDB: 5FBC; [13]; Fig. 15B).

Kovaľ *et al*. [13] reported two binding modes of nucleotides – deep (W_NBS1_ is replaced by a part of the nucleobase) and shallow (nucleobase is in interaction with W_NBS1_ in the NBS1 site), with only the latter being suspected to be consistent with cleavage. In the SmNuc1 structures, we observed the deep binding mode only in the case of 5′‐GMP in SmNuc1:GMP. This is the first observation of the deep binding mode which is in accordance with the reaction mechanism and demonstrates again the broad ability of S1/P1 nucleases to remodel the active site.

### Cleavage of c‐di‐GMP

In Husťáková *et al*. [5], a significant activity (with rate ~ 220 s^−1^) of SmNuc1 towards cyclic diguanosine‐5′‐monophosphate (c‐di‐GMP), the bacterial second messenger, was reported. This activity was in this study confirmed by a crystallographic experiment when c‐di‐GMP was soaked into a crystal of SmNuc1, resulting in the SmNuc1:GMP* structure with 5′‐GMP bound as a product of c‐di‐GMP cleavage. Several residues involved in ligand binding (Arg74, Asn161 and Lys158) are relatively disordered (visible on ellipsoids of anisotropic atomic displacement parameters, ADPs), and the active site is less stabilised compared to the SmNuc1:GMP structure. During the catalytic process, it is inevitable that an intermediate state (pGpG) of c‐di‐GMP cleavage is unbinding and rebinding, in which these less stabilised residues are likely to be involved. SmNuc1:GMP* differs slightly from SmNuc1:GMP due to the absence of phosphate (Pi) in the active site, which is replaced by water molecules. Arg74 is also not used to stabilise the ribose moiety of the ligand due to the intermediate state of the Arg74‐motif.

### Additional binding sites of SmNuc1 and comparison to other S1/P1 nucleases

**Fig. 16:**
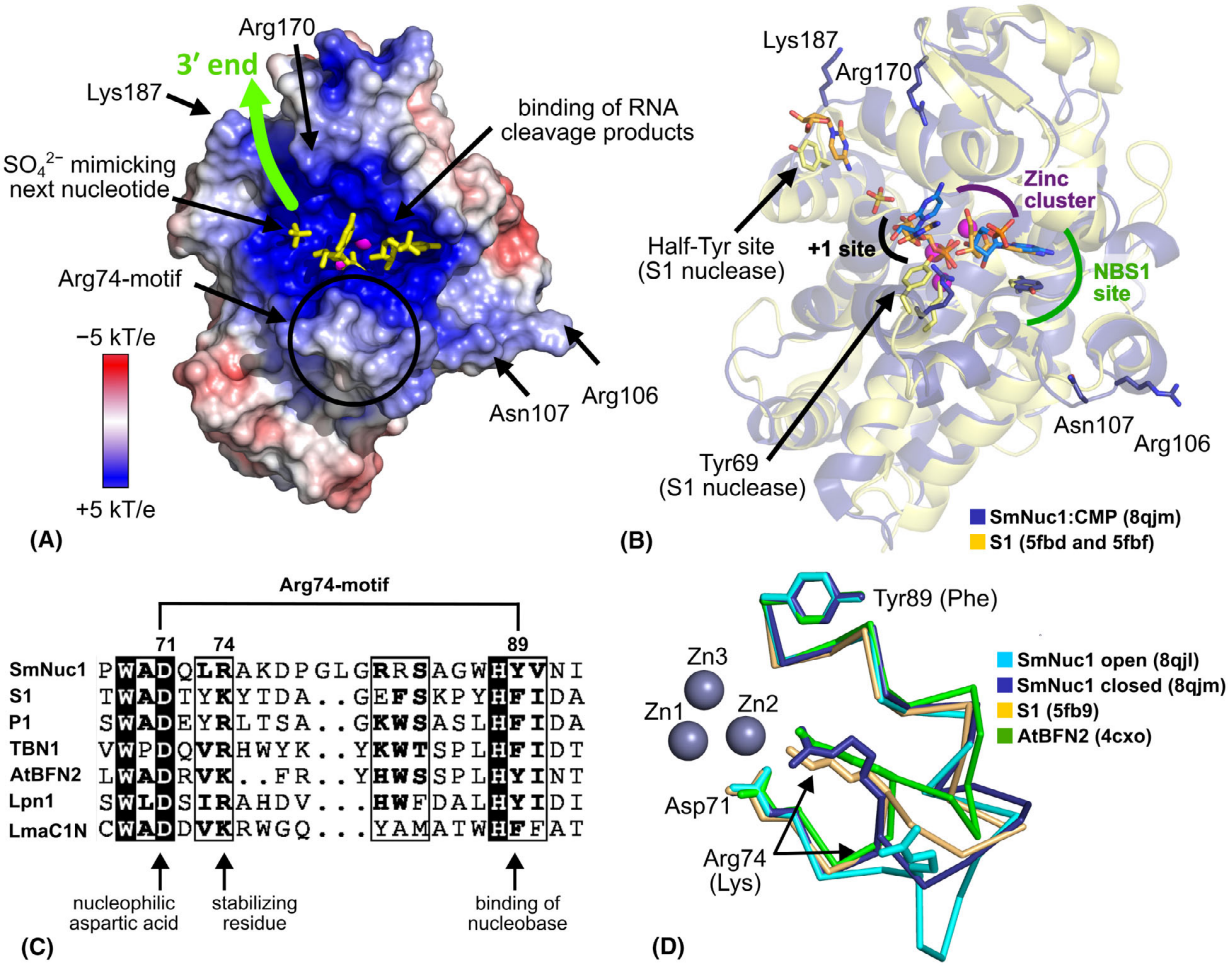
Binding of nucleic acids to SmNuc1 and comparison of the Arg74‐motif within known structures of the S1/P1 family nucleases. (A) SmNuc1 surface coloured by electrostatic potential (−5 to +5 kT·e^−1^) with predicted binding of the substrate (green arrow) on the SmNuc1 nuclease surface. This model is based on binding of ligand in the SmNuc1:CMP complex (yellow sticks). Zinc ions are displayed as magenta spheres. Residues Lys187, Arg170, Asn107 and Arg106 potentially involved in binding of longer substrates are marked. Electrostatic potential was calculated using the apbs software [46]. (B) The superposition of SmNuc1 nuclease in the complex with 5′‐CMP (PDB: 8QJM) and assembly of S1 nuclease structures (PDB entries 5FBF and 5FBD, [13]) in complexes with 5′‐dCMP ligands. SmNuc1 is shown as blue cartoon with Arg74 and Tyr89 shown as sticks. 5′‐CMP molecules are displayed as sticks (C atoms in blue). S1 nuclease is shown as yellow cartoon with important residues Tyr183 (Half‐Tyr site), Tyr69 (stabilisation of +1 site), Lys68 and Phe81 (substrate/product binding) as yellow sticks. Nucleotides 5′‐dCMP bound to S1 nuclease are shown as sticks (C in orange). (C) Sequence alignment of the SmNuc1 Arg74‐motif within the S1/P1 family. Amino acid sequences of several nucleases from the family were chosen: SmNuc1 from *Stenotrophomonas maltophilia* (GenBank: WP_005410840.1), S1 from *Aspergillus oryzae* (UniProt: P24021), P1 from *Penicillium citrinum* (UniProt: P24289), TBN1 from *Solanum lycopersicum* (UniProt: Q0KFV0), AtBFN2 from *Arabidopsis thaliana* (UniProt: Q9C9G4), Lpn1 from *Legionella pneumophila* (UniProt: Q5ZV70) and LmaC1N from *Leishmania major* (UniProt: Q8T4M4). The alignment was calculated in *Clustal Omega* [41] and visualised in espript 3.0 (https://espript.ibcp.fr, [47]). (D) Superposition of the Arg74‐motif (Asp71–Tyr89) of SmNuc1 in the open position (light blue, PDB: 8QJL) with SmNuc1 in the closed position (dark blue, PDB: 8QJM), S1 nuclease (yellow, PDB: 5FB9, [13]), and AtBFN2 nuclease (dark green, PDB: 4CXO, [14]). Zinc ions are shown as grey spheres. The alignments were calculated, and all graphics were created using pymol (Schrödinger, LLC).

In SmNuc1:CMP, the position of two nucleotides in the active site of SmNuc1 captures the state of the nucleolytic cycle immediately after the cleavage of the phosphodiester bond (Fig. 5B). This is a similar position to the second binding mode of the phosphate ion (Pi) in the structure of S1 nuclease [13]. The sulphate ion (${\mathrm{SO}}_{4}^{2-}$) in this structure close to the +1 position of the active site possibly mimics the position of the phosphate moiety of the next residue (+2 position). Accordingly, we can propose the binding of a longer RNA chain to SmNuc1 which is in agreement with the proposed ssDNA binding to S1 nuclease [13] (Fig. 16A,B).

Similarities and differences in other binding sites of SmNuc1 compared to other S1/P1 nucleases with different substrate preferences may then be important clues to possible dsDNA binding. In P1 and S1 nucleases, there is an additional binding site on the surface far from the catalytic zinc cluster (the Tyr site in P1 and the Half‐Tyr site in S1 nuclease; [13, 21]), which was not found in the case of SmNuc1. At the corresponding position in SmNuc1, there are no aromatic residues accessible for binding of nucleobase of a longer nucleic acid chain, but the surface area in the place of these sites contains positive residues (e.g. Arg170 and Lys187, Fig. 16A,B), which makes this environment more similar to that of TBN1 nuclease [22]. The activity of SmNuc1 and TBN1 towards dsDNA substrates is 6900 and 3900 U·μg^−1^, respectively [5, 23], whereas it is significantly lower in the case of the fungal P1 and S1 nucleases [13, 24]. In TBN1, a finger‐like binding site (Gln105/His106), has also been proposed, to be involved in binding of dsDNA substrate [22]. In SmNuc1, the Arg106/Asn107 pair at the end of the active site groove (~ 20 Å from Zn3) may have a similar function (Fig. 16A). No other additional binding sites were identified.

### Arg74‐motif and its role in SmNuc1 activity

SmNuc1 retains 15% of its activity against RNA at 10 °C [5] pointing to similar properties (low temperature activity, high catalytic efficiency, easy large‐scale production) as observed for cold‐active enzymes [25]. *S. maltophilia* has already been isolated from cold water samples and tubes [26], and several cold‐active enzymes produced by *Sm* have been studied [27, 28]. SmNuc1 has an unusually high activity towards all substrates as documented by the *k*_cat_ values for the 3′‐nucleotidase activity for SmNuc1, TBN1 and Lpn1 of 4100, 320, and 390 s^−1^, respectively, showing a 10‐fold difference (calculations based on previously reported activities [5, 23, 29]).

The observed flexibility of the motif containing Arg74 is unique in the known structures of the S1/P1 nuclease family. This corresponds well to the cold‐active character of this nuclease. The Arg74‐motif consists of important catalytic residues. It starts with residue Asp71, which is essential for nucleophilic attack by the water molecule [4]. The motif ends with the aromatic residue Tyr89, which is part of NBS1 and binds nucleobases by π–π stacking. The third important residue is Arg74, the positively charged residue close to the active site, which is a stabilising residue for substrates and products during the cleavage process. The importance of this residue was experimentally confirmed by our mutational study but also by mutational studies of S1 (mutant Lys68Asn decreased its activity towards RNA to 20% and towards ssDNA to 40%, [13]) and also of TBN1 (methylation of Lys73 decreased its activity to 60% towards dsDNA, [22]).

**Fig. 17:**
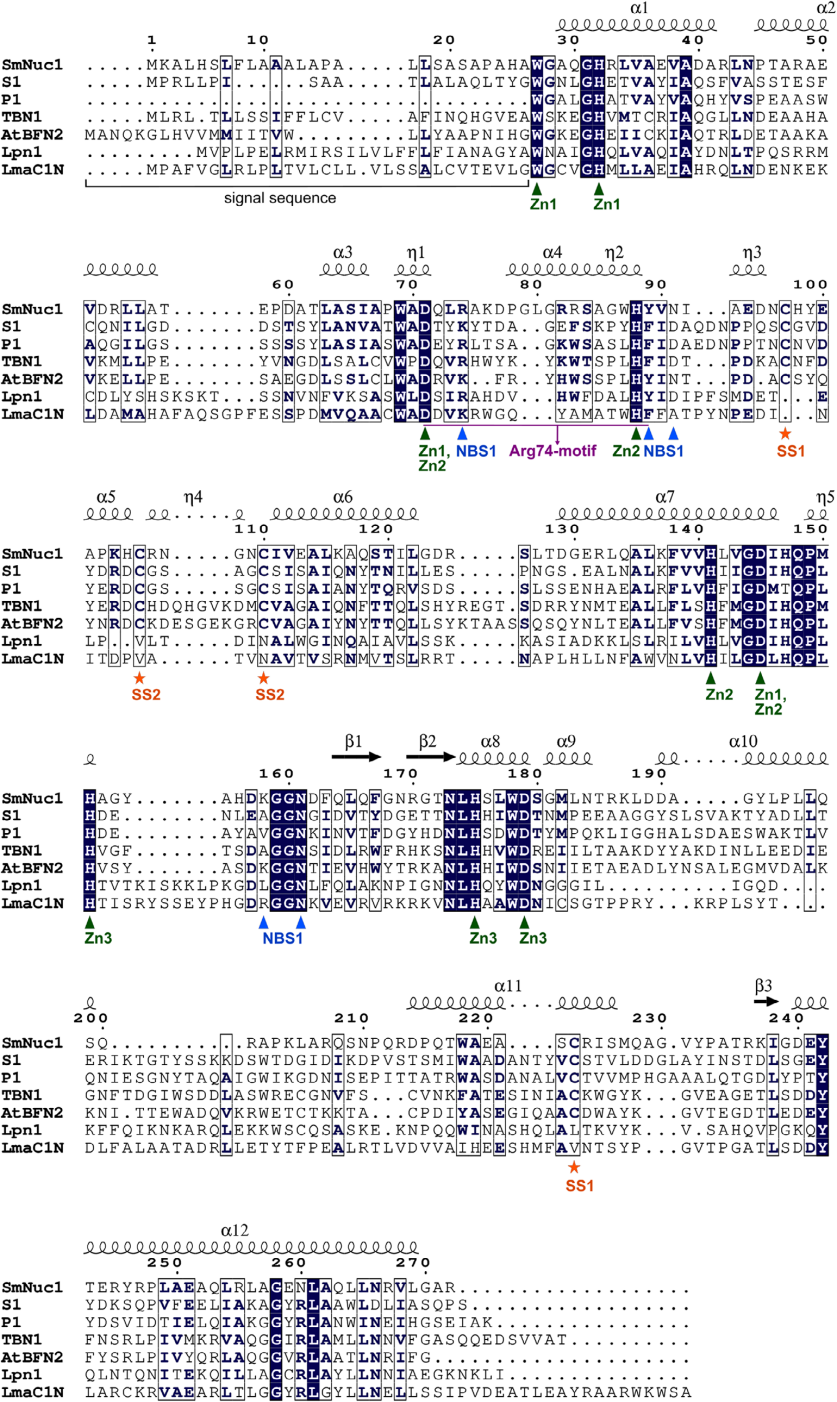
Sequence alignment of SmNuc1 with selected S1/P1 nucleases. SmNuc1 from *Stenotrophomonas maltophilia* (GenBank: WP_005410840.1), S1 from *Aspergillus oryzae* (UniProt: P24021), P1 from *Penicillium citrinum* (UniProt: P24289), TBN1 from *Solanum lycopersicum* (UniProt: Q0KFV0), AtBFN2 from *Arabidopsis thaliana* (UniProt: Q9C9G4), Lpn1 from *Legionella pneumophila* (UniProt:Q5ZV70), and LmaC1N from *Leishmania major* (UniProt entry Q8T4M4). The secondary structure elements of SmNuc1 are shown above the alignment. Conserved residues are marked by dark blue background, partially conserved or similar residues are in dark blue bold letters, and globally similar residues are in boxes. The Zn‐binding, NBS1 residues, and the Arg74‐motif are marked. Disulphides of SmNuc1 are marked as SS1 and SS2. The alignment was calculated in clustal omega [32] and visualised in espript 3.0 (https://espript.ibcp.fr, [33]).

The whole motif from the nucleophile‐coordinating aspartic acid to the nucleobase‐binding aromatic residue is in the case of SmNuc1 longer by two amino acids than the same part in the case of S1 (UniProt: P24021; [13]), P1 (UniProt: P24289; [21]) or TBN1 (UniProt: Q0KFV0; [22]). It is three amino acids longer than in the Lpn1 (UniProt: Q5ZV70; [29]) or the LmaC1N (UniProt: Q4Q7F4; [30]) nucleases and even four amino acids longer than in the AtBFN2 nuclease (UniProt: Q9C9G4; [14]). The number and type of amino acids in this motif presumably allow for the Arg74‐motif mobility in SmNuc1 and could contribute to the high catalytic rate of this enzyme (Figs 16C,D and 17).

In SmNuc1, we observed Arg74 in several positions involved in ligand binding, in particular stabilising products immediately after the cleavage process in both the NBS1 (−1) and the +1 site (Fig. 5A). The mobility of the entire region containing Arg74 may therefore be involved in the product leaving step of the catalytic cycle when the movement facilitates the process. An acceleration of the process can be one of the reasons for the increased activity rate of SmNuc1 compared to other S1/P1 nucleases [5].

In our previous study of S1 nuclease [16], the most mobile residues in the active site were described and predicted to be involved in the catalytic cycle. Our studies of SmNuc1 confirmed the mobility of those important residues with relatively higher flexibility in comparison to S1 nuclease and new flexible features of the active site, such as Lys158 and the whole Arg74‐motif (Fig. 16).

### Selectivity towards RNA and DNA substrates

When Arg74 was mutated to Gln, we observed a decrease in activity on DNA substrates (both ssDNA and dsDNA), although the activity towards RNA substrate was maintained or even slightly increased. Similarly, the mutation to Lys only affected activity on DNA substrates, but with a smaller decrease than in the case of Gln, probably due to only a minor change in charge distribution. We hypothesise that RNA substrates/products are stabilised by O2′ contacts to other parts of the active site through water molecule W_O2′_ and that Arg74 is therefore not essential for RNA binding. In addition, it is possible that the absence of this positively charged residue leads to an easier and faster release of products, thereby increasing the rate of activity against RNA compared to SmNuc1WT.

Naturally, S1/P1 nucleases keep the ability to cleave both DNA and RNA and tend to be rather single‐strand specific and sugar non‐specific, but with the mutation of this single amino acid the substrate preference was significantly shifted towards RNase. For SmNuc1WT, the ratio of activity towards RNA and ssDNA substrate is 1.5 : 1 according to Husťáková *et al*. [5]. For SmNuc1R74Q, this ratio has shifted to approximately 6 : 1 (Fig. 14). To the best of our knowledge, it is the first case, when S1/P1 nuclease was optimised towards one type of substrate.

### RNA cleavage and product leaving

**Fig. 18:**
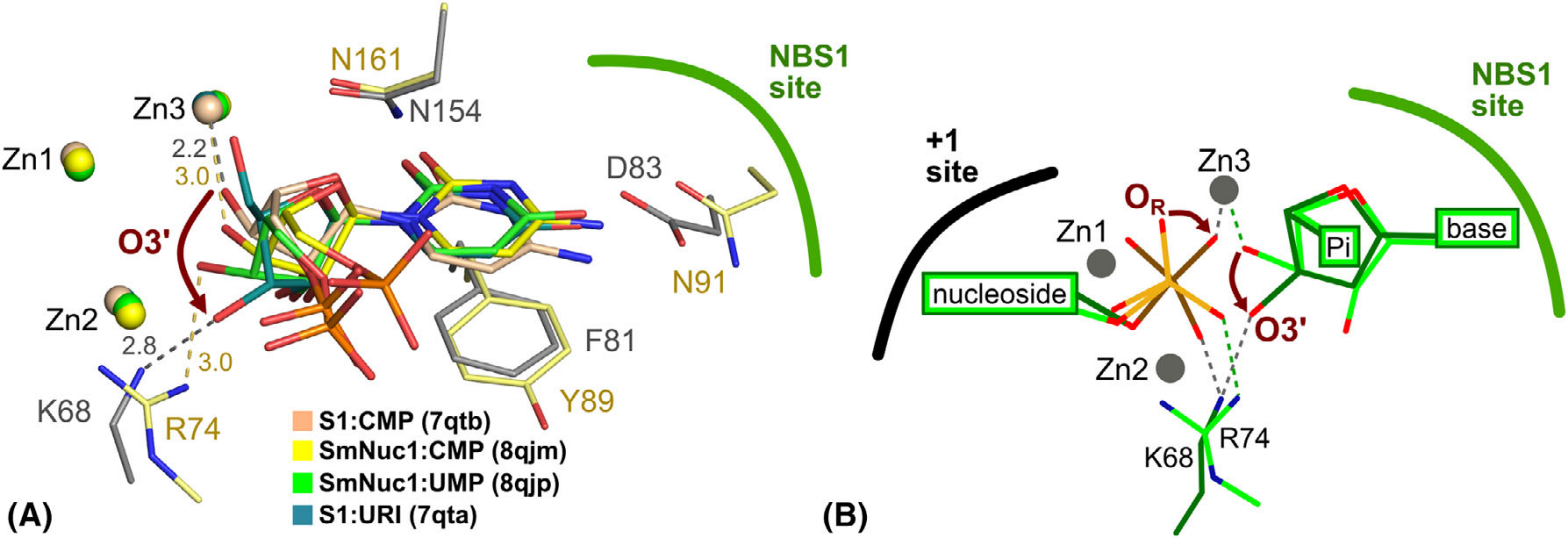
Product‐leaving states of S1/P1 nucleases. (A) The rotation of O3′ oxygen from the active site zinc cluster. A comparison of RNA cleavage products bound to S1 (PDB: 7QTA and PDB: 7QTB; [16]) and SmNuc1 nuclease (PDB: 8QJM and PDB: 8QJP). O3′ is firstly in contact with Zn3 and then moves out, ending up in contact with a positively charged (stabilising) residue Arg74 (Lys68 in S1 nuclease). The active site residues are shown as sticks (C atoms in light yellow for SmNuc1 nuclease, C atoms in dark grey for S1 nuclease), zinc ions are coloured according to ligands. (B) A schema of the cooperative movement of the phosphate group and O3′ of products in the active site. The O3′ oxygen of the nucleotide in the NBS1 site rotates out from the zinc cluster and the oxygen O_R_ of the nucleotide in +1 position rotates (50°) and takes a shallower binding pose with respect to the zinc cluster. The scheme is based on the after‐cleavage state captured in the structure SmNuc1:CMP (C in light green, P in yellow) and the product‐leaving state in the structure of S1 nuclease in complex with 5′‐dCMP (C in dark green, P in brown, PDB: 5FBF, [13]). All graphics and alignments were done using pymol (Schrödinger, LLC).

Structures of SmNuc1 with several products reported here cover and at the same time confirm several states of the RNA cleavage cycle (described in Ref. [4]). After the binding of the substrate to the active site of the enzyme, the nucleophilic water molecule present between Zn1 and Zn2 (Fig. 2A) attacks the phosphate moiety which connects the O5′ of the nucleotide in the +1 position and O3′ of the nucleotide in the NBS1 site and causes its inversion, which results in the P‐O3′ bond cleavage. Two resulting products are captured in the SmNuc1:CMP structure (PDB: 8QJM). O3′ of the ribose moiety of the nucleotide in the NBS1 site begins to rotate out of the zinc cluster in the active site. Similarly, the nucleotide in the +1 position rotates and the phosphate moiety releases itself from the zinc cluster and leaves the active site. Together with the structures of S1 nuclease in complexes with RNA products S1:URI (PDB: 7QTA) and S1:CMP (PDB: 7QTB; both [16]), we have captured four states of the O3′ motion (Fig. 18A), suggesting that this change is likely not a one‐time flip but a gradual process. We suggest that to maintain the distance between the involved oxygens, the rotation of the nucleotide in the +1 position and the O3′ movement of the nucleotide in the NBS1 site are cooperative. This hypothesis is also supported by the observation of an intermediate state with rotated nucleotide in the +1 site and nucleotide in the NBS1 site with O3′ flipped out in the structure of S1 nuclease in the complex with 5′‐dCMP (PDB: 5FBF, [13]) (Fig. 18B).

## Conclusion

We report here the first structural study of a bacterial S1/P1 nuclease, SmNuc1 from an opportunistic pathogen *S. maltophilia*, with RNA cleavage products, uncovering the key features of the enzyme. The substrate preference of S1/P1 nucleases has previously not been fully understood. SmNuc1 is an RNA‐preferring member of the family, for which the degree of preference can be significantly increased, via a single mutation, favouring RNA.

SmNuc1 possesses a unique flexible Arg74‐motif which plays a role in the substrate binding and product release and is likely the cause of the unusually high catalytic rate. At the same time, Arg74 of this motif has been shown to discriminate between RNA and DNA as substrate. Mutation of Arg74 to Gln has led to a 4‐fold increase of the RNase/ssDNase activity ratio. This mobile element may also contribute to the observed activity at low temperatures.

For the first time, purine nucleotide binding in the substrate/product position was observed and the previously suggested details of the catalytic mechanism based on pyrimidine binding patterns are also confirmed for purines. The 5′‐AMP and 5′‐GMP binding modes are not identical, which illustrates the adaptability of NBS1 of the S1/P1 family.

The rate‐limiting step of catalysis is most likely the product unbinding, for which we bring new insights. The observed tight interactions between the phosphate group of the +1 nucleotide and ribose of the −1 nucleotide imply a mechanistic cooperation of products in the departure from the active site.

The direct observation of the c‐di‐GMP cleavage product *in crystallo* confirms the activity of SmNuc1 in the crystalline form, implying several steps of binding, cleavage, release and rebinding realised in the crystal.

The structural explanation of the high activity, proven optimization of substrate selectivity and broad temperature tolerance make SmNuc1 a highly attractive enzyme for biotechnology applications.

## Materials and methods

### Expression and purification of SmNuc1 wild type

SmNuc1 wild type (SmNuc1WT) was expressed and purified as previously published [5]. Briefly, the *SmNuc1* gene (GenBank: WP_005410840.1) with MalE signal peptide was cloned into the Champion™ pET303/CT‐His vector (Invitrogen, Thermo Fisher Scientific, Waltham, MA, USA). The resulting *SmNuc1WT* vector was transformed into *Escherichia coli* Lemo21(DE3) cells (New England Biolabs, Ipswich, MA, USA) via a heat shock protocol. Expression was carried out overnight in the autoinduction Overnight Express™ Instant TB Medium (Novagen, Merck, Darmstadt, Germany) with 50 μg·mL^−1^ carbenicillin and 30 μg·mL^−1^ chloramphenicol at 30 °C to OD_600_ = 0.6 and then the temperature was lowered to 20 °C. Protein purification from the bacterial lysate was performed by Ni^2+^ chelate affinity chromatography (Ni‐NTA) using a HisTrap™ FF column (GE Healthcare, Cytiva, Marlborough, MA, USA) and an ÄKTA Purifier (GE Healthcare). The next step was heparin affinity chromatography. After dialysis using the Mini Dialysis Kit (1 kDa cut‐off, 2 mL; GE Healthcare) against binding buffer (50 mm Bis‐Tris, 100 mm NaCl, pH 6.6), the protein was applied to HiTrap™ Heparin HP column (GE Healthcare) and eluted by gradient elution with the same buffer containing 1 m NaCl. The resulting SmNuc1 fractions were detected spectrophotometrically at 280 nm and analysed by 12% SDS/PAGE. The purest fractions were desalted and concentrated using Microsep™ Advanced centrifugation devices (3 kDa cut‐off; Pall Corporation, Cytiva, Marlborough, MA, USA) and stored in the storage buffer (50 mm Tris, 150 mm NaCl, pH 7.5) at 4 °C.

### Selecting, cloning and expression of mutated variants

Mutated variants were prepared using a restriction‐free PCR cloning protocol according to Unger *et al*. [31]. Forward primers with incorporated point mutation at residue R74 (5′‐GACCAGCTGAAGGCAAAGG‐3′ for R74K and 5′‐GACCAGCTGCAAGCAAAGGA‐3′ for R74Q; Generi Biotech, Hradec Králové, Czech Republic) and T7 reverse primer (5′‐GCTAGTTATTGCTCAGCGG‐3′; Addgene, Watertown, MA, USA) were diluted to a concentration of 10 μm in nuclease‐free water (NFW; New England Biolabs). The 50 μL PCR mixture for target amplification contained 0.5 μL of *SmNuc1WT* vector as DNA template, 2.5 μL R74 forward primer, 2.5 μL T7 reverse primer, 25 μL Q5® High‐Fidelity 2× Master Mix (New England Biolabs) and 19.5 μL NFW (New England Biolabs), and the PCR was performed according to the manufacturer's instructions.

Products of the PCR (R74K‐T7rev and R74Q‐T7rev megaprimers, ~ 800 bp length) were extracted from agarose gel using the QIAquick® Gel extraction kit (Qiagen, Hilden, Germany). Vector *SmNuc1WT* DNA was linearized using XhoI restriction endonuclease (New England Biolabs) according to the manufacturer's instructions (reaction mixture contained 3 μL XhoI, 10 μL *SmNuc1WT* DNA, 3 μL FD buffer, and 15 μL NFW; 2 h, 25 °C) and purified using the PCR Purification Kit – Column Kit (Jena Bioscience, Jena, Germany). The final cloning reaction mixture contained 20 ng of linearized *SmNuc1WT* DNA, 100 ng of R74K‐T7rev or R74Q‐T7rev megaprimer, 25 μL Q5® High‐Fidelity 2× Master Mix (New England Biolabs) supplemented with NFW to 50 μL. The PCR thermocycling conditions were initial denaturation at 98 °C for 3 min, 45 cycles at 98 °C for 30 s, 60 °C for 30 s, 72 °C for 8 min, and a final extension at 72 °C for 2 min. Ten microlitres of the reaction mixture without further purification was mixed with 50 μL *E. coli* DH5α competent cells and transformed using a heat shock protocol. The transformed cells were grown on the LB agar plates with 50 μg·mL^−1^ carbenicillin overnight. Separate colonies were inoculated into 10 mL of LB media containing 50 μg·mL^−1^ carbenicillin and plasmids *SmNuc1R74Q* and *SmNuc1R74K* were then isolated using the QIAGEN Plasmid Mini Kit (Qiagen). Incorporation of the point mutation was verified by DNA sequencing (using primers for T7 promoter and T7 terminator).

Expression and purification of mutant variants was performed using the same protocol as described above and monitored by SDS/PAGE.

### Confirmation of SmNuc1 mutants by mass spectrometry

Protein samples were firstly reduced with dithiothreitol and alkylated with iodoacetamide and then digested with trypsin. The cleaved peptides were further analysed on an Agilent 1200 liquid chromatography system (Agilent Technologies, Santa Clara, CA, USA) with timsToF Pro PASEF mass spectrometer (operated in positive mode) and Captive spray (Bruker Daltonics, Billerica, MA, USA). Data processing was performed using the peaksstudio 10.0 software (Bioinformatics Solutions, Waterloo, ON, Canada) with the following search parameters: enzyme – trypsin (specific), carbamidomethylation as a fixed modification, oxidation of methionine and acetylation of protein N terminus as variable modifications.

### Crystallisation and diffraction data measurement

**Fig. 19:**
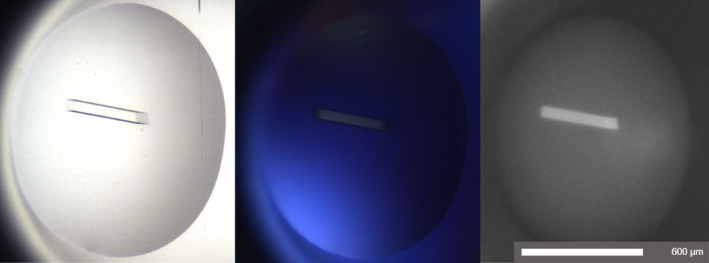
The first crystal of SmNuc1 nuclease, leading to the structure of SmNuc1:free (PDB: 8QJL). The crystal is shown in visible light, polarised light and UV light (from left to right). Images were captured by an RI1000 (Formulatrix).

The SmNuc1WT sample for the initial crystallisation trials was in storage buffer and had a concentration of 10 mg·mL^−1^. Using commercially available kits, crystallisation screening was set up using the Gryphon crystallisation robot (Art Robbins Instruments, Sunnyvale, CA, USA) and the protein:reservoir ratios of 2 : 1, 1 : 1, 1 : 2 in 0.3 μL drops. 96‐well crystallisation plates were stored in a crystallisation hotel (RI1000; Formulatrix, Bedford, MA, USA) at 20 °C. A single crystal (with the longest dimension of ~ 400 μm; Fig. 19) was obtained in the condition No. 74 of the Index™ screen (Hampton Research, Aliso Viejo, CA, USA) (0.1 m Bis‐Tris pH 5.5, 25% w/v PEG 3350, 0.2 m Li_2_SO_4_) after 2 days in drop with a protein : reservoir ratio of 1 : 1.

More crystals were obtained from manually set crystallisation plates using the sitting and hanging drop vapour diffusion techniques in four crystallisation conditions (0.1 m Bis‐Tris pH 5.5, 25% w/v PEG 3350, 0.2 m Li_2_SO_4_; 0.1 m MES pH 6.5, 0.2 m (NH_4_)_2_SO_4_, 30% w/v PEG 8000; 0.1 m MES pH 6.5, 0.2 m Li_2_SO_4_, 30% w/v PEG 8000 and 0.1 m MES pH 6.5, 0.2 m (NH_4_)_2_SO_4_, 30% w/v PEG 5000) in 3–7 days, and protein at concentration 7.5–12 mg·mL^−1^. The ratio of protein to reservoir was 1 : 1 or 1 : 2 and the drop volumes ranged from 0.4 to 2.0 μL. As a cryoprotectant 20% and 25% v/v glycerol (SmNuc1:free, SmNuc1:UMP, SmNuc1:AMP, SmNuc1:GMP, SmNuc1:CMPinh, SmNuc1:GMP*), or 30% v/v PEG 400 (SmNuc1:CMP) in the respective reservoir solutions were used. In order to obtain complexes with products of RNA cleavage, 5′‐adenosine monophosphate (5′‐AMP; Sigma‐Aldrich catalogue No. A1752, Darmstadt, Germany), 5′‐cytidine monophosphate (5′‐CMP; Sigma‐Aldrich catalogue No. C1006), 5′‐guanosine monophosphate (5′‐GMP; Sigma‐Aldrich catalogue No. G8377) and 5′‐uridine monophosphate (5′‐UMP; Sigma‐Aldrich catalogue No. U6375) were dissolved in the corresponding reservoir solution containing cryoprotectant. The final ligand concentration was 20 mm. Cyclic diguanosine‐5′‐monophosphate (c‐di‐GMP; Sigma‐Aldrich catalogue No. SML1228) was dissolved in the corresponding reservoir solution containing cryoprotectant with the final ligand concentration of 5 mm. Crystals were soaked in these solutions for 1–5 min and then vitrified in liquid nitrogen. X‐ray diffraction data were collected either on a D8 Venture diffractometer with a MetalJet liquid metal X‐ray source and a Photon II detector (Bruker AXS GmbH, Karlsruhe, Germany, installed at the Centre of Molecular Structure, Institute of Biotechnology AS CR) in the case of SmNuc1:free and SmNuc1:CMP, synchrotron beamlines 14.1 (SmNuc1:AMP, SmNuc1:GMP, SmNuc1:CMPinh and SmNuc1:GMP*) and 14.2 (SmNuc1:UMP) of the synchrotron radiation source BESSY II, Helmholtz Zentrum Berlin, Germany [32]. Detailed crystallisation and data collection parameters are given in Table 1.

**Table 1:** Crystallisation conditions, data collection, and processing statistics for SmNuc1 structures. Values in parentheses are for the highest resolution shell. Processing statistics as provided by xdsgui [34] and aimless [35]. For SmNuc1:AMP and SmNuc1:GMP* processing statistics are as provided by staraniso [36] and Wilson *B* factor is calculated by the wwPDB validation service [44].

Structure title	SmNuc1:free	SmNuc1:CMP	SmNuc1:AMP	SmNuc1:GMP	SmNuc1:UMP	SmNuc1:CMPinh	SmNuc1:GMP*
PDB ID	8QJL	8QJM	8QJN	8QJO	8QJP	8QJQ	9EMG
Crystallisation condition	0.1 m Bis‐Tris pH 5.5, 25% w/v PEG 3350, 0.2 m Li_2_SO_4_	0.1 m Bis‐Tris pH 5.5, 25% w/v PEG 3350, 0.2 m Li_2_SO_4_	0.1 m MES pH 6.5, 30% w/v PEG 8K, 0.2 m (NH_4_)_2_SO_4_	0.1 m Bis‐Tris pH 5.5, 25% w/v PEG 3350, 0.2 m Li_2_SO_4_	0.1 MES pH 6.5, 30% w/v PEG 8K, 0.2 m Li_2_SO_4_	0.1 m Bis‐Tris pH 5.5, 25% w/v PEG 3350, 0.2 m Li_2_SO_4_	0.1 MES pH 6.5, 30% w/v PEG 5K, 0.2 m (NH_4_)_2_SO_4_
Ligand	–	5′‐CMP	5′‐AMP	5′‐GMP	5′‐UMP	5′‐CMP	c‐di‐GMP
Crystallisation method	Sitting drop vapour diffusion	Sitting drop vapour diffusion	Hanging drop vapour diffusion	Hanging drop vapour diffusion	Hanging drop vapour diffusion	Hanging drop vapour diffusion	Hanging drop vapour diffusion
Protein + reservoir drop volume	0.2 + 0.2 μL	0.5 + 0.5 μL	0.5 + 1.0 μL	0.5 + 0.5 μL	0.5 + 0.5 μL	0.5 + 0.5 μL	1.0 + 1.0 μL
Cryoprotection	20% v/v glycerol	30% v/v PEG 400	20% v/v glycerol	20% v/v glycerol	20% v/v glycerol	25% v/v glycerol	25% v/v glycerol
Diffractometer or beam line	D8 Venture diffractometer (Bruker)	D8 Venture diffractometer (Bruker)	BESSY II BL 14.1	BESSY II BL 14.1	BESSY II BL 14.2	BESSY II BL 14.1	BESSY II BL 14.1
Wavelength (Å)	1.3418	1.3418	0.9184	0.9184	0.9184	0.9184	0.9184
Detector	Photon II	Photon II	Pilatus 6M	Pilatus 6M	Pilatus 2M	Pilatus 6M	Pilatus 6M
Crystal detector distance (mm)	50.0	110.4	321.6	372.3	181.8	267.5	211.4
Rotation range per image (°)	0.5	0.5	0.1	0.1	0.1	0.1	0.1
Exposure time per image (s)	30	30	0.1	0.1	0.1	0.1	0.1
Space group	*P*2_1_2_1_2	*P*2_1_	*P*2_1_	*P*2_1_	*P*2_1_	*P*2_1_	*P*2_1_
*a*, *b*, *c* (Å)	72.5, 73.0, 50.0	43.1, 72.8, 81.2	44.5, 72.7, 82.8	43.2, 73.1, 81.6	44.4, 72.8, 82.9	43.3, 73.2, 81.7	43.3, 72.6, 82.9
α, β, γ (°)	90.0, 90.0, 90.0	90.0, 105.0, 90.0	90.0, 102.7, 90.0	90.0, 105.3, 90.0	90.0, 102.3, 90.0	90.0, 105.0, 90.0	90.0, 102.1, 90.0
Resolution range (Å)	41.2–1.40 (1.42–1.40)	41.66–1.65 (1.68–1.65)	43.385–1.65 (1.69–1.65)	41.64–1.85 (1.89–1.85)	43.41–1.20 (1.22–1.20)	41.78–1.80 (1.84–1.80)	43.36–1.15 (1.18–1.15)
Mosaicity (°)	0.234	0.201	0.268	0.865	0.086	0.307	0.136
*R*_meas_	0.122 (1.320)	0.149 (0.722)	0.113 (0.935)	0.151 (1.538)	0.090 (1.337)	0.117 (1.153)	0.054 (0.701)
Completeness spherical, elliptical (%)	95.9 (98.1), −	99.2 (98.1), −	85.1 (67.6), 88.8 (92.8)	96.4 (99.3), −	97.0 (94.0), −	99.4 (98.9), −	90.6 (55.4), 96.8 (87.9)
Average *I/*σ(*I*)	19.9 (1.6)	6.8 (1.9)	13.2 (2.2)	7.1 (1.1)	11.5 (1.4)	14.2 (1.9)	18.5 (2.8)
CC_1/2_	0.999 (0.498)	0.961 (0.559)	0.998 (0.777)	0.994 (0.374)	0.998 (0.579)	0.995 (0.699)	1.000 (0.824)
Total no. of reflections	728 862 (10 971)	186 687 (8459)	363 710 (18 948)	140 554 (9132)	799 335 (37 635)	303 461 (16 606)	1 085 927 (44 593)
No. of unique reflections	50 815 (2541)	57 878 (2787)	52 701 (2636)	40 335 (2553)	155 893 (7417)	45 358 (2614)	165 597 (8280)
Multiplicity	14.3 (4.3)	3.2 (3.0)	6.9 (7.2)	3.5 (3.6)	5.1 (5.1)	6.7 (6.4)	6.6 (5.4)
Wilson *B* factor (Å^2^)	8.9	5.9	13.7	19.2	11.5	19.5	8.0

### Data processing

SmNuc1:free data were processed using xdskappa [33], scaled using xscale [34], and merged using aimless [35]. Data for SmNuc1:CMP were processed using xdskappa and xdsgui [34], and scaled and merged using aimless. Data for SmNuc1:AMP, SmNuc1:GMP, SmNuc1:UMP, SmNuc1:CMPinh and SmNuc1:GMP* were processed and scaled using xdsgui and merged using aimless.

The diffraction data showed high anisotropy in the case of the SmNuc1:AMP and SmNuc1:GMP* complexes. The largest difference in the suggested diffraction limit (according to the *I*/σ(*I*) > 1.5 criterion) along the main reciprocal lattice directions was 0.6 Å for the SmNuc1:AMP data and 0.4 Å for the SmNuc1:GMP* data as reported by aimless. Therefore, an anisotropy correction was applied to both data using staraniso [36].

The SmNuc1:UMP data suffered from a lattice translocation defect (LTD), as evidenced by non‐origin peaks in the Patterson function and strong peaks in difference electron density at the position of the translocated zinc cluster, similar to our previous work on a closely related enzyme [16]. The datasets were corrected using the formula for one translocation in the crystal [37], where the total observed structure factor $F_{\text{total}}$ is defined as

(1) $$F_{\text{total}}=\left[k+\left(1-k\right)\times \exp\left(2\pi iht_{d}\right)\right]\times F_{\text{unit}}$$

where *k* is a fraction of untranslated layers, $\left(1-k\right)$ is a fraction of translated crystal layers, **h** is reciprocal vector (*h*, *k*, *l*) and $t_{d}$ is the specific translocation vector. The intensity formula is then

(2) $$I_{\text{total}}=\left[\left(2k^{2}-2k+1\right)+2k\times \left(1-k\right)\times \cos\left(2\pi ht_{d}\right)\right]\times I_{\text{unit}}$$

The final high‐resolution cut‐off for the SmNuc1:free, SmNuc1:GMP, SmNuc1:UMP, SmNuc1:CMPinh structures was determined using the paired refinement protocol in pairef [38].

### Structure solution and refinement

The phase problem was initially solved for SmNuc1:free by the molecular replacement method with molrep [39] from the ccp4 suite [40] using a modified structure of S1 nuclease (PDB: 5FB9; [13]) with 29.3% sequence identity (calculated by clustal2.1, [41]) as a template. The molecular replacement solution parameters were relatively weak with low ${\mathrm{CC}}_{F_{o},F_{c}}$ = 0.3284 and high *R*_work_ = 0.636, but with a clearly resolved and localised active site zinc cluster. The structure was manually rebuilt using coot [42] and after several refinement cycles using refmac5 [43] *R*_work_ decreased to 0.12. The phase problem for all other structures was solved by molecular replacement with molrep using the SmNuc1:free structure as the search model.

All structures were built and analysed in coot [42] and refined in refmac5 [43]. A test set of 5% of reflections was excluded during refinement for cross‐validation and then used in the final refinement cycle. Anisotropic atomic displacement parameters (ADPs) were used in the refinement of the SmNuc1:free, SmNuc1:UMP and SmNuc1:GMP*. Hydrogen atoms were refined in riding positions. The geometric restraints for ligands with chemical component dictionary identifiers C5P (cytidine‐5′‐monophosphate), U5P (uridine‐5′‐monophosphate), AMP (adenosine‐5′‐monophosphate), G5P (guanosine‐5′‐monophosphate) and GOL (glycerol) were generated using the Grade web server (http://grade.globalphasing.org/cgi‐bin/grade/server.cgi). The quality of the structures was verified using the validation tools provided by the wwPDB validation service [44] and those implemented in coot. Ligands present in the structures were verified against 2m*F*_o_ – D*F*_c_ composite omit maps (Figs 3, 6, 7, and 9), which were generated in phenix [45] using the refinement option. The final refinement parameters are summarised in Table 2.

**Table 2:** The structure parameters and refinement statistics for SmNuc1 structures. Structure‐refinement parameters and statistics are as provided by refmac5 [43]. 1PE, pentaethylene glycol; PEG, diethylene glycol; PGE, triethylene glycol.

Structure title	SmNuc1:free	SmNuc1:CMP	SmNuc1:AMP	SmNuc1:GMP	SmNuc1:UMP	SmNuc1:CMPinh	SmNuc1:GMP*
PDB ID	8QJL	8QJM	8QJN	8QJO	8QJP	8QJQ	9EMG
Resolution (Å)	1.40	1.65	1.65	1.85	1.20	1.80	1.15
*R*_work_	0.1186	0.1726	0.1747	0.1714	0.1339	0.1663	0.1120
*R*_free_	0.1612	0.1979	0.2143	0.2091	0.1669	0.2025	0.1314
*R*_all_	0.1228	0.1732	0.1759	0.1723	0.1369	0.1675	0.1129
Average *B* factor (Å^2^)	13.9	12.2	19.0	28.0	20.5	29.1	15.2
Overall figure of merit	0.908	0.910	0.855	0.802	0.855	0.869	0.930
Average ${\mathrm{CC}}_{F_{o},F_{c}}$	0.9552	0.9042	0.8714	0.8813	0.9384	0.9140	0.9427
R.m.s.d. bond lengths from ideal (Å)	0.0112	0.0106	0.0105	0.0082	0.0101	0.0076	0.0093
R.m.s.d. bond angles from ideal (°)	1.6920	1.6073	1.5690	1.4451	1.5679	1.4082	1.6064
Ramachandran favoured^a^ (%)	97.75	97.83	98.2	97.0	98.0	96.6	97.97
Ramachandran outliers^a^ (%)	0.00	0.00	0.00	0.00	0.00	0.00	0.00
Asymmetric unit content	1× SmNuc1 3× Zn^2+^ 3× ${\mathrm{SO}}_{4}^{2-}$ 2× glycerol 455× water	2× SmNuc1 6× Zn^2+^ 3× 5′‐CMP 1× 1PE 1× Na^+^ 13× ${\mathrm{SO}}_{4}^{2-}$ 538× water	2× SmNuc1 6× Zn^2+^ 1× 5′‐AMP 1× ${\mathrm{PO}}_{4}^{3-}$ 3× ${\mathrm{SO}}_{4}^{2-}$ 1× glycerol 589× water	2× SmNuc1 6× Zn^2+^ 1× 5′‐GMP 2× PEG 1× PGE 1× Na^+^ 1× Cl^−^ 2× ${\mathrm{PO}}_{4}^{3-}$ 19× ${\mathrm{SO}}_{4}^{2-}$ 1× glycerol 379× water	2× SmNuc1 6× Zn^2+^ 2× 5′‐UMP 1× ${\mathrm{PO}}_{4}^{3-}$ 3× ${\mathrm{SO}}_{4}^{2-}$ 6× glycerol 721× water	2× SmNuc1 6× Zn^2+^ 2× 5′‐CMP 1× PEG 1× 1PE 20× ${\mathrm{SO}}_{4}^{2-}$ 2× glycerol 418× water	2× SmNuc1 6× Zn^2+^ 1× 5′‐GMP 1× PEG 3× glycerol 7× ${\mathrm{SO}}_{4}^{2-}$ 747× water

^a^ According to the wwPDB validation service [44].

The coordinates and structure factors of the SmNuc1:free, SmNuc1:CMP, SmNuc1:AMP, SmNuc1:GMP, SmNuc1:UMP, SmNuc1:CMPinh and SmNuc1:GMP* structures have been deposited in the PDB as entries 8QJL, 8QJM, 8QJN, 8QJO, 8QJP, 8QJQ, and 9EMG, respectively. Diffraction images have been deposited in the Structural Biology Data Grid (https://data.sbgrid.org/) under the following identifiers https://doi.org/10.15785/SBGRID/1063 for SmNuc1:free, https://doi.org/10.15785/SBGRID/1064 for SmNuc1:CMP, https://doi.org/10.15785/SBGRID/1065 for SmNuc1:AMP, https://doi.org/10.15785/SBGRID/1066 for SmNuc1:GMP, https://doi.org/10.15785/SBGRID/1067 for SmNuc1:UMP, https://doi.org/10.15785/SBGRID/1068 for SmNuc1:CMPinh, and https://doi.org/10.15785/SBGRID/1082 for SmNuc1:GMP*.

### Nuclease activity assay

Nuclease activity of SmNuc1WT and mutated variants was measured against dsDNA, ssDNA and RNA. Calf thymus DNA (Sigma‐Aldrich catalogue No. D3664) and RNA from yeast (Sigma‐Aldrich catalogue No. R6625) were dissolved in reaction buffer (50 mm Tris pH 7.5, 50 mm NaCl). Single‐stranded DNA was prepared by heat‐denaturation of dsDNA. The enzymatic reaction mixture with a total volume of 100 μL contained SmNuc1 samples (0.1 μg·mL^−1^ in the case of ssDNA and RNA, 1 μg·mL^−1^ in the case of dsDNA) and 200 μg·mL^−1^ ssDNA, dsDNA, or RNA. Reactions were incubated at 37 °C for 5 min in the case of RNA and ssDNA substrates and for 10 min in the case of dsDNA. Reactions were stopped by adding 250 μL of isopropanol. The reaction mixtures were vortexed and incubated at −20 °C for 30 min. Undigested precipitated substrate was sedimented by centrifugation at 20 000 ***g*** for 40 min at 4 °C and the absorbance of the supernatant was measured at 260 nm. One unit of nuclease activity was defined as change of absorbance of 0.001 in 1 cm path per 1 min [23]. All enzymatic reactions were performed in triplicate (including background measurements).

## Conflict of interest

The authors declare no conflict of interest.

## Author contributions

KA, JDo, TK, MT planned experiments; KA, JDu, BH prepared samples; KA, TK, MT, PK, BH performed experiments; KA, JDo, TK, MT wrote paper with contributions from other co‐authors; KA, TS, JDo, TK corrected manuscript.

### Peer review

The peer review history for this article is available at https://www.webofscience.com/api/gateway/wos/peer‐review/10.1111/febs.17265.

## Data Availability

The measured data are available in the Structural Biology Data Grid (https://data.sbgrid.org/) under identifier https://doi.org/10.15785/SBGRID/1063 for SmNuc1:free, https://doi.org/10.15785/SBGRID/1064 for SmNuc1:CMP, https://doi.org/10.15785/SBGRID/1065 for SmNuc1:AMP, https://doi.org/10.15785/SBGRID/1066 for SmNuc1:GMP, https://doi.org/10.15785/SBGRID/1067 for SmNuc1:UMP, https://doi.org/10.15785/SBGRID/1068 for SmNuc1:CMPinh, and https://doi.org/10.15785/SBGRID/1082 for SmNuc1:GMP*. The structural data are available in the wwPDB at https://doi.org/10.2210/pdb8qjl/pdb for SmNuc1:free; https://doi.org/10.2210/pdb8qjm/pdb for SmNuc1:CMP, https://doi.org/10.2210/pdb8qjn/pdb for SmNuc1:AMP, https://doi.org/10.2210/pdb8qjo/pdb for SmNuc1:GMP, https://doi.org/10.2210/pdb8qjp/pdb for SmNuc1:UMP, https://doi.org/10.2210/pdb8qjq/pdb for SmNuc1:CMPinh, and https://doi.org/10.2210/pdb9emg/pdb for SmNuc1:GMP*.
